# Technology, attributions, and emotions in post-secondary education: An application of Weiner’s attribution theory to academic computing problems

**DOI:** 10.1371/journal.pone.0193443

**Published:** 2018-03-12

**Authors:** Rebecca Maymon, Nathan C. Hall, Thomas Goetz, Andrew Chiarella, Sonia Rahimi

**Affiliations:** 1 Department of Educational & Counselling Psychology, McGill University, Montreal, Quebec, Canada; 2 Department of Empirical Educational Research, University of Konstanz, Konstanz, Germany; 3 Thurgau University of Teacher Education, Kreuzlingen, Switzerland; 4 Educational Psychology, Athabasca University, Athabasca, Alberta, Canada; Public Library of Science, UNITED KINGDOM

## Abstract

As technology becomes increasingly integrated with education, research on the relationships between students’ computing-related emotions and motivation following technological difficulties is critical to improving learning experiences. Following from Weiner’s (2010) attribution theory of achievement motivation, the present research examined relationships between causal attributions and emotions concerning academic computing difficulties in two studies. Study samples consisted of North American university students enrolled in both traditional and online universities (total *N* = 559) who responded to either hypothetical scenarios or experimental manipulations involving technological challenges experienced in academic settings. Findings from Study 1 showed stable and external attributions to be emotionally maladaptive (more helplessness, boredom, guilt), particularly in response to unexpected computing problems. Additionally, Study 2 found stable attributions for unexpected problems to predict more anxiety for traditional students, with both external and personally controllable attributions for minor problems proving emotionally beneficial for students in online degree programs (more hope, less anxiety). Overall, hypothesized negative effects of stable attributions were observed across both studies, with mixed results for personally controllable attributions and unanticipated emotional benefits of external attributions for academic computing problems warranting further study.

## Introduction

Technology use and attitudes toward computing in education are becoming increasingly examined in educational psychology research, particularly on student development in post-secondary education [1,2]. Considering the prevalence of computing within education, pedagogical use of information communication technology (ICT) is arguably now an essential cornerstone of personalized learning in teacher education [3]. In a recent investigation of ICT use in European higher education, motivation and learning strategies were found to predict educational platform use, highlighting the importance of examining students’ psychological responses to educational computing issues [4]. Recent research has also examined the impact of teaching technologies on learning outcomes [5], with student motivation and attitudes having been studied in relation to game-based e-learning [6], computer-based assessment [7], and social media as educational tools [8]. However, despite academic technology now supporting much of student learning in higher education (e.g., requisite software, web-based tools, online courses; see [9]), there exists a largely unaddressed need to support digital literacy to optimize student success [2].

Learning technology has been shown to facilitate university students’ self-directed learning, particularly at post-secondary institutions with large student-to-faculty ratios. Nevertheless, whereas research by Deepwell and Malik [10] found that up to 80% of students viewed technology as important to their learning, and over 60% indicated positive effects of learning technology on their attitudes toward independent study, findings also showed frequent requests for guidance on how to use specific educational technologies. Moreover, some research shows students to not view learning technology as effective in enhancing their learning experiences, including recent work by McCabe and Meuter [11] examining business students’ perceptions of course management software. Technological difficulties have also been linked to problems with learning, performance, and attrition in online continuing education programs [12], with learners who reported higher pre-enrollment motivation levels being less likely to drop out following technological difficulties. Such findings highlight the need for further research on the effects of motivational variables on how post-secondary students respond to technological challenges in both online and traditional settings so as to inform computer-assisted instruction (CAI) as well as educational technology support (e.g., e-learning [1]; Web 2.0 resources [13]).

With respect to instructional technology, motivational predictors of adaptive responses to academic computing issues have to date been explored mainly in terms of technology-related self-efficacy, anxiety, interest, and user experience [14–17]. Beyond these general concepts, motivation research investigating students’ interpretations as to why computer problems occur and how these explanations impact their educational technology experiences is lacking. Further, despite a growing importance on the specific nature of students’ emotional experiences in research on web-based educational settings (e.g., [18]), relevant research has primarily explored the effects of students’ attitudes regarding educational technology. Accordingly, the present studies address a need for theory-informed research that more specifically examines students’ motivational and affective responses to computing challenges so as to contribute to the critical developing literature on the psychological impact of technological opportunities and difficulties in educational settings.

## Causal attributions in educational settings

### Weiner’s attribution theory

According to Weiner’s [19–21] attribution theory, an individual engages in causal search following success and failure events, with failure eliciting greater causal search. Outcomes that are important, negative, or unexpected are proposed to elicit greater causal search resulting in an attribution that can be classified according to three properties: locus, controllability, and stability. *Locus* refers to whether an individual believes the cause of the success or failure was internal or external to themself, *controllability* reflects one’s beliefs concerning the extent to which the cause was volitional in nature, and *stability* reflects one’s belief as to how likely the cause is to change over time. Attribution theory further hypothesizes that these causal explanations, in turn, influence individuals’ subsequent emotions and behaviors. In this model, attribution-dependent emotions include locus-related emotions such as pride, feelings of hopefulness following from perceived instability, as well as emotions such as anger, guilt, and shame linked to perceptions of uncontrollability.

More specifically, feelings of guilt are proposed to stem from attributions indicating perceived personal control over one’s failure experience, whereas failure attributions that are internal to the individual but personally uncontrollable should result in feelings of shame. Moreover, feelings of guilt, unlike shame, have been identified as an activating emotion in motivation research [22]. Additionally, feelings of hopelessness are linked to failure experiences for which the cause is believed to be stable over time, whereas hopefulness is assumed to follow from failure attributions to temporally unstable factors [19]. For example, a student who believes they failed an exam due to a lack of ability (likely perceived as personally uncontrollable and stable over time; cf. a fixed theory of intelligence [23]) is likely to experience hopelessness regarding future tests. Conversely, a student who perceives a failing grade as due to a lack of effort should feel guilty yet also hopeful due to corresponding attribution characteristics of personal controllability and temporal instability typically associated with effortful behavior (cf. incremental self-theories).

### Empirical findings

Empirical research following from Weiner’s [21] theory has consistently found controllable and temporally unstable attributions for academic difficulties to be most beneficial for motivation, persistence, and achievement in post-secondary students. More specifically, university students are repeatedly found to report perceptions of control over their effort and strategy use as potential causes of midterm grades [24], with these types of attributions (vs. uncontrollable attributions) leading to more positive and less negative emotions [25], higher grades [26–27], and lower course withdrawal [28]. Similarly, attributions for poor post-secondary performance to factors that change over time (e.g., lack of familiarity) have been found to correspond with greater expectations for success as well as more positive anticipatory emotions (greater hope, lower anxiety; e.g., [25,29–30]. Recent findings also support the hypothesized precursors to causal search in university students, with both scenario and “in vivo” studies by Stupnisky, Stewart, Daniels, and Perry [31] showing that although negative (vs. positive) academic outcomes were the strongest predictors of causal search, outcomes that were also unexpected, or both unexpected and important, further contributed to explaining when students engaged in causal search.

Although little research has examined the effects of college students’ attributions for academic computing experiences, with relevant computing research having focused on K-12 experiences (e.g., [32–35]), some studies do suggest potential links between attributions for computing difficulties and post-secondary learning and achievement. For example, findings with college students show qualitative differences between internal and external attributions for web search effectiveness [36], that ability attributions for failure in a computer course correlate with lower enrolment [37], and the prevalence of attributions to controllable factors (e.g., practice) in computer programming courses [38]. Findings with undergraduates further show positive relationships between external and stable attributions for computer course grades and anxiety, stable and internal attributions and course grades, and between external attributions and computer experience [39]. Students who make external attributions for computing problems have also been found to report greater computer experience in experimental research involving computer tasks [40].

Research on computing-related attributions has also examined gender stereotypes, following from scenario research typically showing female university students to make more maladaptive attributions for computing failures compared to their male counterparts [41]. For example, findings from an experimental study by Koch et al. [40] with German students (ages 16–21) showed females to make more internal attributions, and males more external attributions, for hardware malfunctions, with females also reporting less computer use, self-efficacy, and knowledge relative to males. A similar study with German students by Sieverding and Koch [42] on attributions to ability and luck for completing a computer task showed that although females did not demonstrate gender bias when evaluating the performance of others (i.e., attributed success to ability vs. luck for both genders), they nonetheless evaluated their own computer competence as lower than males due to gender stereotypes.

## The present research

Although some prior research on computing-related attributions provides preliminary empirical support for the application of Weiner’s [21] attribution theory to the academic computing domain, there are limitations that warrant further research. More specifically, most available findings are based on hypothetical or scenario measures [41] and outcomes involving qualitative attribution categorization, computer experience, course enrolment, or achievement, warranting further experimental study [40] and evaluation of students’ emotions as critical outcomes [18]. Additionally, further research examining online vs. traditional student samples is needed to better address student experiences with online learning tools [10–11]. As technology mediates the learning experiences of students participating in online programs more pervasively than those enrolled in traditional in-class programs, exploration of how these two groups of students differentially experience academic computing problems is warranted to inform institution-specific efforts for computing-related student support. Finally, given that the measurement of attributions in prior research has often not assessed the critical attributional dimension of perceived controllability [40] or combined across multiple attributions types (i.e., ability vs. luck [42]), research utilizing causal dimensions of attributions as per Weiner’s [21] attribution theory is needed to better understand the relationships between students’ attributions and emotions specific to academic computing.

To provide a more comprehensive understanding of how university students’ causal attributions influence their emotions with respect to academic computing issues, the present research examined these variables with both experimental and scenario methods across two studies with university students in traditional as well as online learning environments. More specifically, the present studies assessed students’ attributions and emotions specific to academic computing difficulties by contrasting results from hypothetical scenarios with those from experimentally manipulated computing failures. Incorporating recent research on the precursors to causal search [31], this research also examined the effects of causal attributions as a function of varying degrees of causal search as elicited by manipulations of the expectedness and importance of a computing failure experience in an effort to better identify not only emotionally adaptive attribution types, but also the types of situations in which the effects of causal attributions on emotions are most evident.

## Ethics statement

Protocols for the present studies were approved by the Research Ethics Boards of McGill University and Athabasca University. All studies were conducted according to the ethical principles expressed in the WMA Declaration of Helsinki. Participants were presented with an electronic informed consent form before beginning each of the online studies, for which continuation onto the first page of the study indicated consent to participate. Participants were instructed to print a copy of the consent form for their records. This electronic form of consent to participate in the present studies was approved by each of the aforementioned ethics boards. Participation in all studies was voluntary, unnecessary deception was avoided, and all analyses were conducted on anonymous data as identifiers were removed following data collection and entry.

## Study hypotheses

Based on Weiner’s [21] attribution theory, prior experimental research on causal search in academic settings [31], as well as existing literature, the following hypotheses concerning the effects of causal attributions on emotions were examined.

Hypothesis 1. More internal (i.e., less external) attributions for technology-related problems should predict more positive and less negative emotions concerning academic computing. Similarly, attributions specifically to external factors (i.e., perceived as controlled by others) were expected to predict poorer emotional outcomes. In other words, it was anticipated that some attributions would be rated as more external than others (e.g., luck vs. computer quality), with greater externality predicting poorer emotions. No specific hypotheses were proposed for differences between traditional vs. online students due to lack of research contrasting the relationships between attributions and emotions between these populations.

Hypothesis 2. Personally controllable attributions for technology-related problems should better predict more positive and less negative emotions concerning academic computing than the other types of attributions. More specifically, causal attributions that were perceived by students as more personally changeable (e.g., strategy) were expected to predict more adaptive emotions than those perceived as affording less personal control in computing contexts (e.g., luck).

Hypothesis 3. Stable attributions for technology-related problems should predict less positive and more negative emotions concerning academic computing. Following from Weiner’s [21] theory, attributions to factors perceived by students as stable over time were expected to be emotionally maladaptive compared to attributions to factors that could change over time (temporally unstable), as the computing problem would not be expected to improve.

Hypothesis 4. The aforementioned effects of the causal attribution dimensions should be most evident in conditions where the computing failure experience is both *unexpected* and *important*. According to Weiner’s [21] attribution theory, events that are negative, unexpected, and important elicit the most causal search, with the causal attributions selected under such conditions having the greatest impact on subsequent emotions. Conversely, the emotional effects of causal attributions should be least evident in conditions eliciting the least amount of causal search, namely those in which the computing failure is both *expected* and *unimportant*.

## Study 1: Scenario method

### Methods

#### Traditional students

The first study sample was recruited from traditional, in-person university courses in February and March of 2014 and consisted of 144 undergraduates enrolled at a research-intensive North American university. Participants’ mean age was 20.23 years (*SD* = 2.17) and 75.00% were female. Participants’ average self-reported final high school grade was 88.38% (*SD* = 5.73), 72.20% spoke English as a first language, and most were first- or second-year students (Year 1/2/3/4: 30.60%/38.20%/18.80%/11.80%). Faculty affiliations were as follows: 35.40% arts, 30.60% science, 22.20% education, 9.00% other disciplines, and 2.80% in a combined arts and science program.

#### Online students

The second study sample was recruited from a large, online North American university between April and July of 2014 and consisted of 178 undergraduates. Participants’ mean age was 33.17 years (*SD* = 9.67) and 80.30% were female. Participants’ average self-reported final high school grade was 80.82% (*SD* = 8.95), 88.80% spoke English as a first language, and most were first-year students (Year 1/2/3/4/5+: 36.50%/18.00%/19.70%/13.50%/11.20%). Faculty affiliations were as follows: 52.20% humanities and social sciences, 12.90% business, 12.40% health disciplines, 7.30% science and technology, and 14.00% other disciplines.

#### Procedures

Students were recruited by email (both samples) and in person (traditional students) to complete a 20 min questionnaire on academic computing issues, with compensation for participation provided for each sample by being entered into a draw for one of two cash prizes of $250. After completing the demographic and attribution measures, participants read one of four randomly assigned scenarios followed by the emotion measures and a debriefing page. In each scenario condition, participants read one of four hypothetical vignettes involving an academic computing problem and completed emotion measures concerning their likely reaction to the scenario. The four scenarios represented combinations of two causal search dimensions–expected vs. unexpected computing failures, and important vs. unimportant occurrences–as per the two critical precursors to causal search following failure proposed in Weiner’s [21] theory (see also [31]).

1. Expected/High Importance. “You have just finished a project for class and the program you are using suddenly crashes. A copy of your work was not saved. This is expected because you have had the same problem with this program before. This project is worth 40% your final grade and you will have to redo your work before the deadline” (Traditional/Online *n*s = 38/38).
2. Expected/Low Importance. “You have just finished a project for class and the program you are using suddenly crashes. A copy of your work was not saved. This is expected because you have had the same problem with this program before. This project is worth 5% your final grade and you will have to redo your work before the deadline” (Traditional/Online *n*s = 36/44).
3. Unexpected/High Importance. “You have just finished a project for class and the program you are using suddenly crashes. A copy of your work was not saved. This is unexpected because you have never had this problem with this program before. This project is worth 40% your final grade and you will have to redo your work before the deadline” (Traditional/Online *n*s = 37/51).
4. Unexpected/Low Importance. “You have just finished a project for class and the program you are using suddenly crashes. A copy of your work was not saved. This is unexpected because you have never had this problem with this program before. This project is worth 5% your final grade and you will have to redo your work before the deadline” (Traditional/Online *n*s = 33/45).

#### Study measures

Descriptive statistics for the causal dimension, emotion, and computing experience measures including scale means, standard deviations, ranges, and internal reliability levels for both the traditional and online samples are provided in Table 1.

**Table 1:** Study 1: Descriptive statistics.

	*Traditional Students*				*Online Students*			
	*M*	*SD*	Range	α	*M*	*SD*	Range	α
*Covariates*								
Age	20.23	2.17	18–35	-	33.17	9.67	18–63	-
Computer experience	3.97	0.74	2–5	-	3.96	0.82	1–5	-
*Attributions*								
Locus of causality	10.02	5.87	3–27	.81	9.89	6.15	3–27	.82
Personal control	14.24	7.95	3–27	.91	13.86	7.99	3–27	.88
Stability	10.98	5.82	3–27	.55	10.16	5.36	3–27	.39
External control	11.29	6.58	3–27	.78	13.80	7.28	3–27	.84
*Emotions*								
Hope	2.68	2.30	1–10	-	3.65	2.73	1–10	-
Guilt	4.54	3.21	1–10	-	4.60	3.21	1–10	-
Helplessness	6.44	2.85	1–10	-	5.33	3.18	1–10	-
Pride	1.42	1.63	1–10	-	1.35	1.19	1–10	-
Shame	3.73	2.90	1–10	-	3.55	2.93	1–10	-
Enjoyment	1.07	0.60	1–8	-	1.23	1.21	1–10	-
Anxiety	7.88	2.55	1–10	-	7.28	2.89	1–10	-
Boredom	2.11	2.21	1–10	-	1.93	2.02	1–10	-

**Causal attributions:** Causal attributions were assessed using a modified version of the Revised Causal Dimension Scale (CDSII) [43] that assessed students’ perceived locus of causality, personal control, stability, and external control concerning specific attributions for academic computing failure experiences. Participants were asked to first indicate a primary cause for a problem they had encountered while using a computing device (e.g., tablet, laptop, desktop, smartphone, etc.) for academic purposes (e.g., error messages, “freezing,” crashing, etc.) and then to rate the identified cause with respect to four causal dimensions (scale items were additionally modified to use more direct first-person phrasing, such as “myself” vs. “yourself”). Each causal dimension was evaluated with three 9-point items and included locus of causality (internal vs. external to oneself; e.g., 1 = *“The cause reflects an aspect of the situation”*; 9 = *“The cause reflects an aspect of myself”*), personal control (e.g., 1 = *“The cause is something over which I have no power”*; 9 = *“The cause is something over which I have power”*), stability over time (e.g., 1 = *“The cause is temporary”*; 9 = *“The cause is permanent”*), and external control (e.g., 1 = *“The cause is something other people cannot regulate”*; 9 = *“The cause is something other people can regulate”*). Although the internal reliability for the stability scale was lower relative to the other measures (α = .54), it was retained given comparable internal reliability in previous research using the CDSII (e.g., α = .70; [44]; α = .68; [43]).

**Outcome and activity emotions:** Five 10-point items (1 = *not at all*, 10 = *very much so*) were used to measure students’ emotions of hope, guilt, helplessness, pride, and shame concerning the outcomes depicted in the preceding hypothetical scenarios; emotions proposed to follow directly from causal attributions in Weiner’s [21] theory. These outcome-related emotion items were derived directly from those previously used to measure students’ emotions concerning academic achievement [25,29]. Three additional items similarly assessed students’ anticipated emotions of enjoyment, anxiety, and boredom concerning the academic computing activities described in the preceding scenario (see [22]).

**Computing experience:** Students’ perceived academic computing experience relative to others was assessed using a single 5-point item (1 = *none*, 5 = *excellent*) to control for prior experience in our main analyses of the study hypotheses.

### Results

#### Preliminary analyses

**Initial differences:** Independent-samples *t*-tests were conducted to determine initial differences in our study measures as a function of gender and sample type. In the traditional student sample, females reported significantly more feelings of helplessness (*M* = 6.77, *SD* = 2.77) compared to males (*M* = 5.57, *SD* = 2.84; *t*(141) = 2.21, *p* = .029, *d* = .43), with a similar albeit marginally significant gender effect found for anxiety, *t*(140) = 1.87, *p* = .064, *d* = .35. In the online student sample, females also reported greater helplessness (*M* = 5.58, *SD* = 3.24; *t*(60.21) = 2.36, *p* = .021, *d* = .42) and anxiety (*M* = 7.55, *SD* = 2.71; *t*(45.57) = 2.27, *p* = .028, *d* = .45) compared to males (*M* = 4.31, *SD* = 2.72; *M* = 6.17, *SD* = 3.34, respectively). ANOVAs did not reveal significant differences in attributions, age, or computing experience between the scenario conditions for the traditional or online student samples, with chi-square tests showing no differences in gender proportions between scenario conditions.

When assessed on the combined sample of scenario study participants, online students were found to report more externally controllable attributions (*M* = 13.80, *SD* = 7.28; *t*(304) = -3.16, *p* = .002, *d* = .36) than traditional students (*M* = 11.29, *SD* = 6.58). Online students also reported lower levels of helplessness (*M* = 5.33, *SD* = 3.18; *t*(315.87) = 3.29, *p* = .001, *d* = .37) and anxiety (*M* = 7.28, *SD* = 2.89; *t*(316.14) = 1.98, *p* = .049, *d* = .22), as well as more hope (*M* = 3.65, *SD* = 2.73; *t*(319.51) = -3.45, *p* = .001, *d* = .38) than traditional students (*M* = 6.44, *SD* = 2.85; *M* = 7.88, *SD* = 2.55; *M* = 2.68, *SD* = 2.30, respectively). The average age of online students was also higher (*M* = 33.17, *SD* = 9.67; *t*(198.69) = -17.32, *p* < .001, *d* = 1.85) than for traditional students (*M* = 20.23, *SD* = 2.17). Given these significant differences between traditional and online students, as well as different organizational structures with respect to institutions and faculties between institutions, these samples were examined separately in the main analyses.

**Correlational analyses:** Correlations between continuous study variables are presented for both the traditional and online student samples in Table 2. In accordance with Weiner’s [21] attribution theory, there was a strong correlation between internal and personally controllable attributions in both samples, with both variables found to correlate negatively with externally controllable attributions for online students. As expected, positive emotions were positively intercorrelated (e.g., pride and enjoyment) as were negative emotions (e.g., anxiety and helplessness), with significant negative correlations found between positive and negative emotions (e.g., hope and helplessness). Interestingly, there were weak yet significant positive correlations between pride and negative emotions (guilt, shame; for similar findings see [45]), and between enjoyment and boredom in the traditional sample, as well as between pride and boredom in the online sample (for more on positive relationships with specific boredom types, see [46]). As computing experience was negatively correlated with helplessness (traditional sample) and positively correlated with hope (online sample), and age was negatively correlated with helplessness and anxiety (online sample), these background measures were additionally included as covariates alongside gender in our main analyses.

**Table 2:** Study 1: Zero-order correlations.

	1	2	3	4	5	6	7	8	9	10	11	12	13	14
1. Internal attributions	-	.61***	.16*	-.25***	.04	.07	-.03	.03	.09	-.04	-.02	-.01	.01	-.01
2. Personally controllable attributions	.66***	-	-.08	-.24**	.15	.09	-.10	.03	.07	-.04	-.03	.06	-.11	.14
3. Stable attributions	.08	-.04	-	-.01	-.07	.13	.16*	.02	.23**	.02	-.06	-.06	.00	.01
4. Externally controllable attributions	-.01	.01	.02	-	.01	.05	.07	.04	.03	.01	.01	.15*	.09	.02
5. Hope	-.02	.14	-.12	.01	-	-.17*	-.20**	.24***	-.17*	.15*	-.33***	.07	-.03	.26***
6. Guilt	.00	-.07	.19*	.14	.01	-	.25***	-.03	.58***	-.13	.21**	.15*	-.04	-.03
7. Helplessness	-.19*	-.22**	.14	.18*	-.27***	.22**	-	-.12	.26***	-.17*	.46***	.11	-.23**	-.05
8. Pride	-.11	-.14	-.11	.00	.12	.20*	.15	-	-.01	.41***	-.09	.18*	.07	-.04
9. Shame	.05	-.01	.16*	.11	-.16	.43***	.17*	.19*	-	-.01	.23**	.12	-.10	.01
10. Enjoyment	.00	-.04	.10	-.02	.19*	.08	.07	.33***	.13	-	-.17*	.00	.04	-.03
11. Anxiety	-.03	-.17*	.02	.06	-.31***	.12	.38***	.08	.24**	.02	-	.12	-.17*	.00
12. Boredom	-.05	.05	.12	.06	.03	.18*	.03	.11	.20*	.21**	.00	-	-.11	-.06
Covariates														
13. Age	.11	.03	-.04	-.01	-.09	-.04	.07	-.04	.00	-.07	.12	.05	-	-.23**
14. Computer experience	.11	.12	-.03	-.09	.14	.03	-.18*	.04	.08	-.12	-.04	.01	-.21**	-

*Note*. Correlations below the diagonal are for traditional students; correlations above the diagonal are for online students.

*p ≤ .05.

**p ≤ .01.

***p ≤ .001

**Scenario condition effects:** For traditional students, MANOVAs revealed significant differences between scenario conditions on emotions, showing students in the *expected/high importance* (*M* = 8.89, *SE* = .40) and *unexpected/high importance* conditions (*M* = 8.49, *SE* = .40) to report more anxiety than those in the *unexpected/low importance* (*M* = 7.34, *SE* = .43) and *expected/low importance* conditions (*M* = 6.80, *SE* = .41; *F*(3, 136) = 5.68, *p* = .001, η_p_^2^ = .11). Additionally, students in the *expected/high importance* condition reported more shame (*M* = 4.92, *SE* = .48) than students in the *unexpected/high importance* (*M* = 3.62, *SE* = .47), *unexpected/low importance* (*M* = 3.28, *SE* = .50), and *expected/low importance* conditions (*M* = 3.20, *SE* = .48; *F*(3, 136) = 2.75, *p* = .045, η_p_^2^ = .057), with a similar albeit marginally significant effect found for pride, *F*(3, 136) = 2.42, *p* = .069, η_p_^2^ = .051. For online students, significant scenario condition effects showed students in the *expected/high importance* (*M* = 7.87, *SE* = .46) and *unexpected/high importance* conditions (*M* = 8.18, *SE* = .40) to report more anxiety than students in the *unexpected/low importance* (*M* = 6.57, *SE* = .43) and *expected/low importance* conditions (*M* = 6.36, *SE* = .44; *F*(3, 169) = 4.67, *p* = .004, η_p_^2^ = .077).

#### Rationale for main analyses

The hypothesized main effects (attributions on emotions) and interaction effects (attributions by scenario conditions on emotions) were evaluated using linear regressions controlling for gender, age, and computing experience. In each analysis, Step 1 evaluated the effects of background variables, the scenario conditions, and attribution dimensions, with Step 2 evaluating the condition by attribution interaction terms. Due to anticipated conceptual and observed empirical overlap between causal attribution dimensions (e.g., internality vs. personal controllability, personal vs. external control), the four attribution dimensions were evaluated independently in separate regression analyses. Scenario conditions were dummy coded with the *expected/low importance* condition as the reference group, as this condition was expected to elicit the least causal search as per Weiner’s [21] attribution theory. Additionally, all continuous variables in main and interaction effects were mean-centered prior to analysis, the traditional and online samples were analyzed separately, and simple slopes analyses were conducted by reverse coding groups found to have significant interaction effects with the reference group. To allow for interpretation of effects involving a nominal moderator, unstandardized coefficients for regressions are reported (see [47–48]).

#### Main analyses

**Traditional students:** With respect to main effects of the attribution dimensions, internal attributions (*B* = -.080, *p* = .053) and personally controllable attributions (*B* = -.073, *p* = .013) predicted less helplessness in Step 1. However, these effects were not significant once the interaction terms were entered in Step 2 (see Table 3 for effects of internal and personally controllable attributions), with a significant Step 2 suppression effect showing externally controllable attributions to predict more anxiety (*B* = .12, *p* = .053) instead observed (see Table 4 for effects of stable and externally controllable attributions).

**Table 3:** Study 1: Hierarchical regression results for internal and personally controllable attributions made by traditional students.

Predictor	Hope	Guilt	Helplessness	Pride	Shame	Enjoyment	Anxiety	Boredom
Internal attributions								
Step 1/2								
Gender	.30/.26	-.94/-.89	-.79/-.78	-.21/-.23	.35/.42	-.02/-.02	-.93/-.99	.64/.66
Age	-.07/-.05	-.07/-.09	.05/.08	-.01/-.01	.00/-.03	-.02/-.02	.18/.23*	.03/.04
Experience	.48/.53	.13/.08	-.47/-.50	.08/.11	.12/.05	-.02/-.02	-.03/.02	-.44/-.49
Attributions	-.01/-.04	.02/.09	-.08*/-.09	-.03/-.04	.02/.10	.00/.00	-.02/-.10	-.03/-.04
U/H	-1.13/-1.18	.11/.06	1.28*/1.21	-.09/-.13	.43/.39	.03/.03	1.92***/1.87***	-.07/-.04
U/L	-.60/-.56	-.42/-.57	-.31/-.56	-.03/.01	-.03/-.21	-.01/-.01	.77/.72	.55/.42
E/H	-1.39/-1.40	.84/.84	.15/.18	.85/.84	1.63/1.65	.21/.21	2.34***/2.36***	.32/.34
*R*^2^	.09/.10	.04/.06	.11*/.14	.08/.09	.07/.08	.03/.03	.17***/.19	.05/.07
Attributions X U/H	/.10	/-.01	/.10	/.06	/-.05	/-.01	/.15	/-.02
Attributions X U/L	/.05	/-.16	/-.19	/.04	/-.20	/.00	/.01	/-.10
Attributions X E/H	/.00	/-.12	/.02	/-.02	/-.13	/-.01	/.12	/.07
Personally controllable attributions								
Step 1/2								
Gender	.14/.22	-.93/-.89	-.67/-.60	-.20/-.23	.37/.40	.02/.01	-.68/-.73	.56/.59
Age	-.06/-.08	-.06/-.07	.03/.02	-.02/-.01	.00/.01	-.02/-.02	.15/.17	.02/.02
Experience	.33/.22	.25/.19	-.42/-.49	.08/.12	.19/.16	-.02/-.02	-.03/.04	-.47/-.50
Attributions	.04/.10	-.03/.03	-.07**/-.06	-.02/-.03	-.01/.01	.00/.00	-.04/-.10	.02/.02
U/H	-1.01/-.95	.06/.07	1.03/1.04	-.13/-.17	.36/.34	.03/.03	1.76**/1.69**	-.11/-.09
U/L	-.44/-.42	-.61/-.58	-.63/-.68	-.05/-.04	-.22/-.22	.01/.02	.61/.57	.53/.51
E/H	-1.18/-1.16	.71/.64	-.17/-.05	.79/.71	1.53/1.52	.20/.19	2.12***/2.14***	.33/.40
*R*^2^	.08/.11	.05/.07	.12**/.15	.08/.10	.07/.08	.03/.04	.17***/.18	.04/.05
Attributions X U/H	/-.05	/.01	/.00	/.05	/.04	/.00	/.08	/-.03
Attributions X U/L	/-.10	/-.07	/-.09	/.02	/-.05	/.00	/.04	/-.02
Attributions X E/H	/-.12	/-.16	/.05	/-.04	/-.07	/-.02	/.11	/.05

*Note*. Experience reflects computer experience compared to others. Attributions refer to attributions for relevant section. U/H = unexpected/high importance, U/L = unexpected/low importance, and E/H = expected/high importance conditions. Unstandardized *B* coefficients and *R*^2^ values are provided for regressions on study measures. Significance of *R*^2^ values indicates two-tailed significance of change from previous step. For gender: 0 = females, 1 = males.

*p ≤ .05.

**p ≤ .01.

***p ≤ .001

**Table 4:** Study 1: Hierarchical regression results for stable and externally controllable attributions made by traditional students.

Predictor	Hope	Guilt	Helplessness	Pride	Shame	Enjoyment	Anxiety	Boredom
Stable attributions								
Step 1/2								
Gender	.30/.28	-.94/-.79	-.85/-.78	-.28/-.24	.37/.49	.01/.00	-.77/-.80	.57/.53
Age	-.09/-.10	-.05/-.06	.04/.03	-.03/-.02	.01/.01	-.02/-.02	.16/.16	.03/.04
Experience	.43/.42	.20/.19	-.54/-.56	.04/.06	.17/.15	-.02/-.03	-.11/-.13	-.44/-.47
Attributions	-.05/-.04	.09/.04	.05/-.04	-.04/.00	.07/.02	.01/.00	.00/-.08	.06/-.05
U/H	-1.30*/-1.30*	.18/.19	1.29*/1.28*	-.13/-.13	.41/.42	.03/.03	1.93***/1.91***	-.10/-.13
U/L	-.80/-.87	-.57/-.58	-.24/-.21	-.02/.00	-.26/-.22	.03/.02	.85/.90	.59/.69
E/H	-1.52**/-1.56**	.83/.88	.20/.23	.85/.89	1.54/1.63*	.21/.19	2.40***/2.38***	.28/.28
*R*^2^	.11*/.14	.08/.12	.11*/.15	.09/.11	.09/.14	.03/.05	.17***/.20	.06/.16**
Attributions X U/H	/.00	/.23	/.17	/-.01	/.18	/.01	/.05	/.04
Attributions X U/L	/-.12	/.12	/.23*	/-.01	/.17	/.00	/.19	/.32***
Attributions X E/H	/.07	/-.07	/.02	/-.09	/-.07	/.03	/.07	/.07
Externally controllable attributions								
Step 1/2								
Gender	.33/.29	-1.20/-1.10	-1.07/-1.11	-.25/-.23	.22/.15	.02/.00	-.83/-.85	.52/.41
Age	-.08/-.08	-.03/-.04	.05/.05	-.02/-.02	.03/.03	-.02/-.02	.16/.17	.03/.03
Experience	.42/.43	.35/.33	-.41/-.40	.05/.03	.29/.34	-.03/-.02	-.04/.00	-.45/-.39
Attributions	.01/.00	.07/-.03	.07/.08	.00/-.03	.04/.12	.00/.00	.02/.12*	.02/.05
U/H	-1.27/-1.32	.27/.22	1.23/1.21	-.12/-.14	.61/.66	.03/.02	1.91***/2.01***	-.14/-.19
U/L	-.75/-.73	-.16/.04	-.10/-.11	.00/-.04	.17/.17	.01/.01	.88/.90	.53/.61
E/H	-1.51/-1.51	.99/.99	.19/.19	.84/.84	1.81/1.81	.21/.21	2.36***/2.37***	.28/.28
*R*^2^	.09/.10	.07/.11	.12*/.12	.07/.08	.09/.10	.03/.03	.17***/.21	.04/.08
Attributions X U/H	/.06	/.14	/.01	/.05	/-.12	/.01	/-.22*	/.02
Attributions X U/L	/.03	/.23*	/-.03	/.01	/-.09	/.00	/-.11	/.00
Attributions X E/H	/-.02	/.03	/-.03	/.06	/-.11	/-.01	/-.12	/-.13

*Note*. Experience reflects computer experience compared to others. Attributions refer to attributions for relevant section. U/H = unexpected/high importance, U/L = unexpected/low importance, and E/H = expected/high importance conditions. Unstandardized *B* coefficients and *R*^2^ values are provided for regressions on study measures. Significance of *R*^2^ values indicates two-tailed significance of change from previous step. For gender: 0 = females, 1 = males.

*p ≤ .05.

**p ≤ .01.

***p ≤ .001

**Fig 1:**
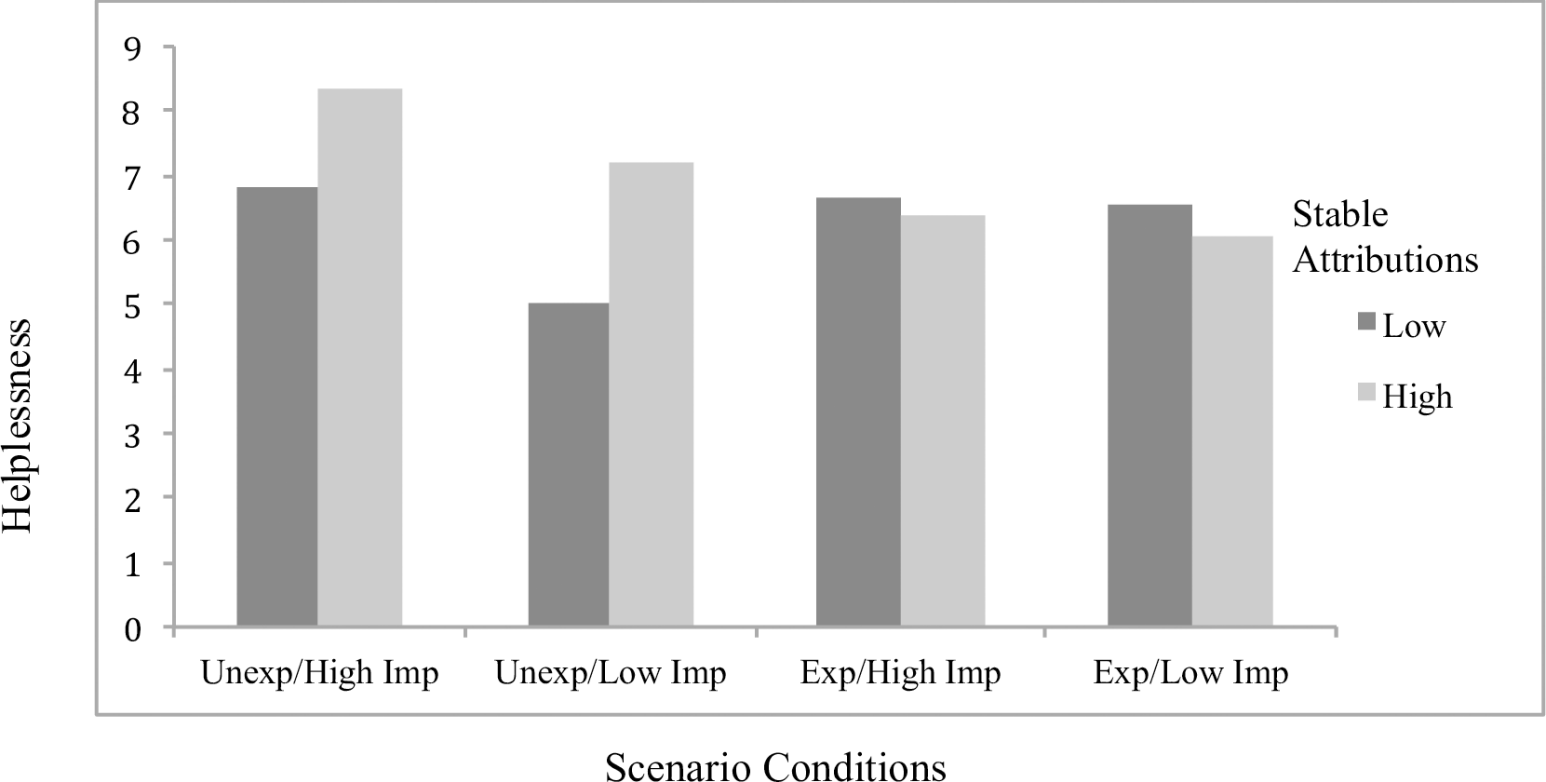
Effects of stable attributions by scenario conditions on helplessness for traditional students.

**Fig 2:**
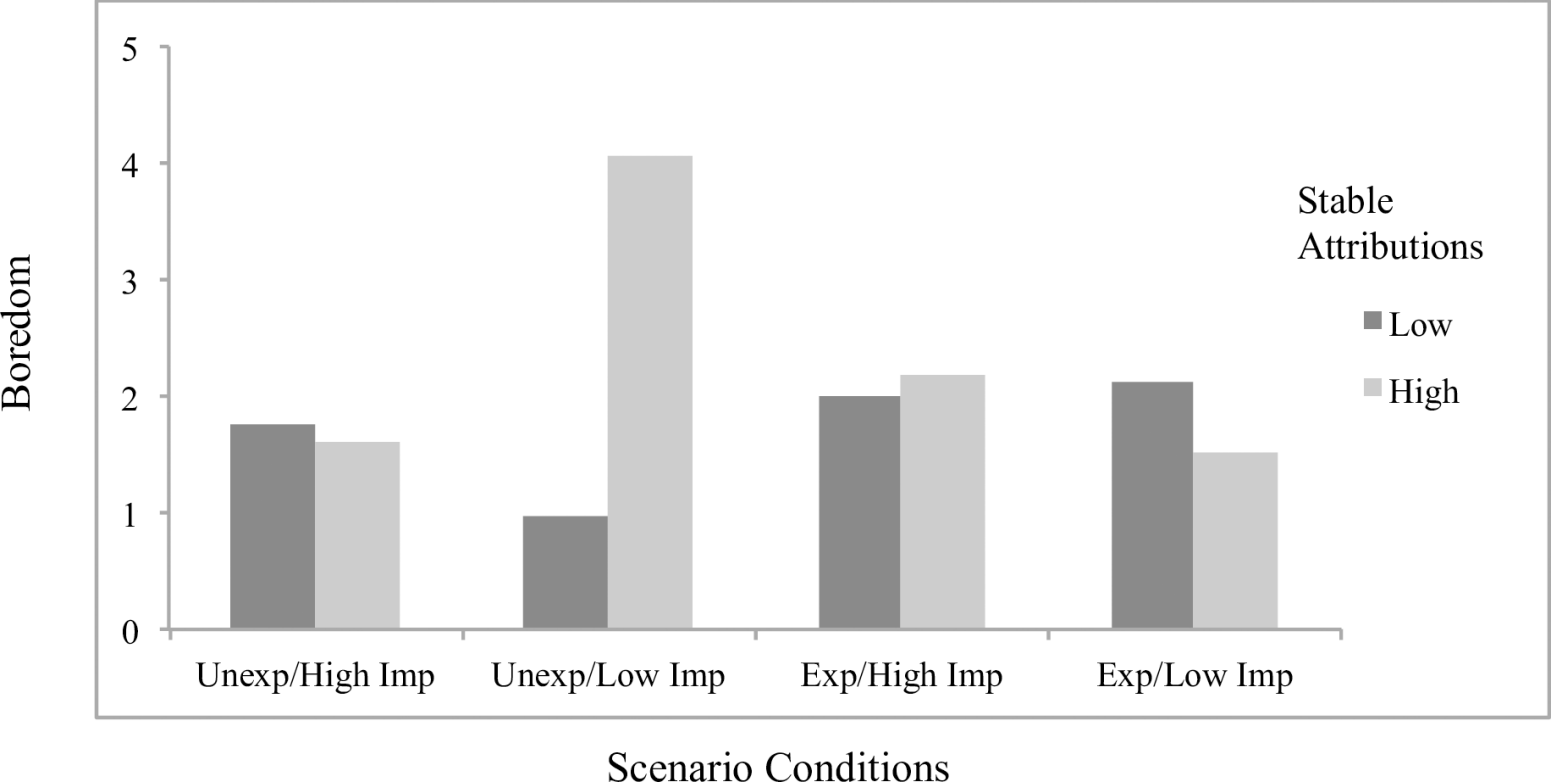
Effects of stable attributions by scenario conditions on boredom for traditional students.

**Fig 3:**
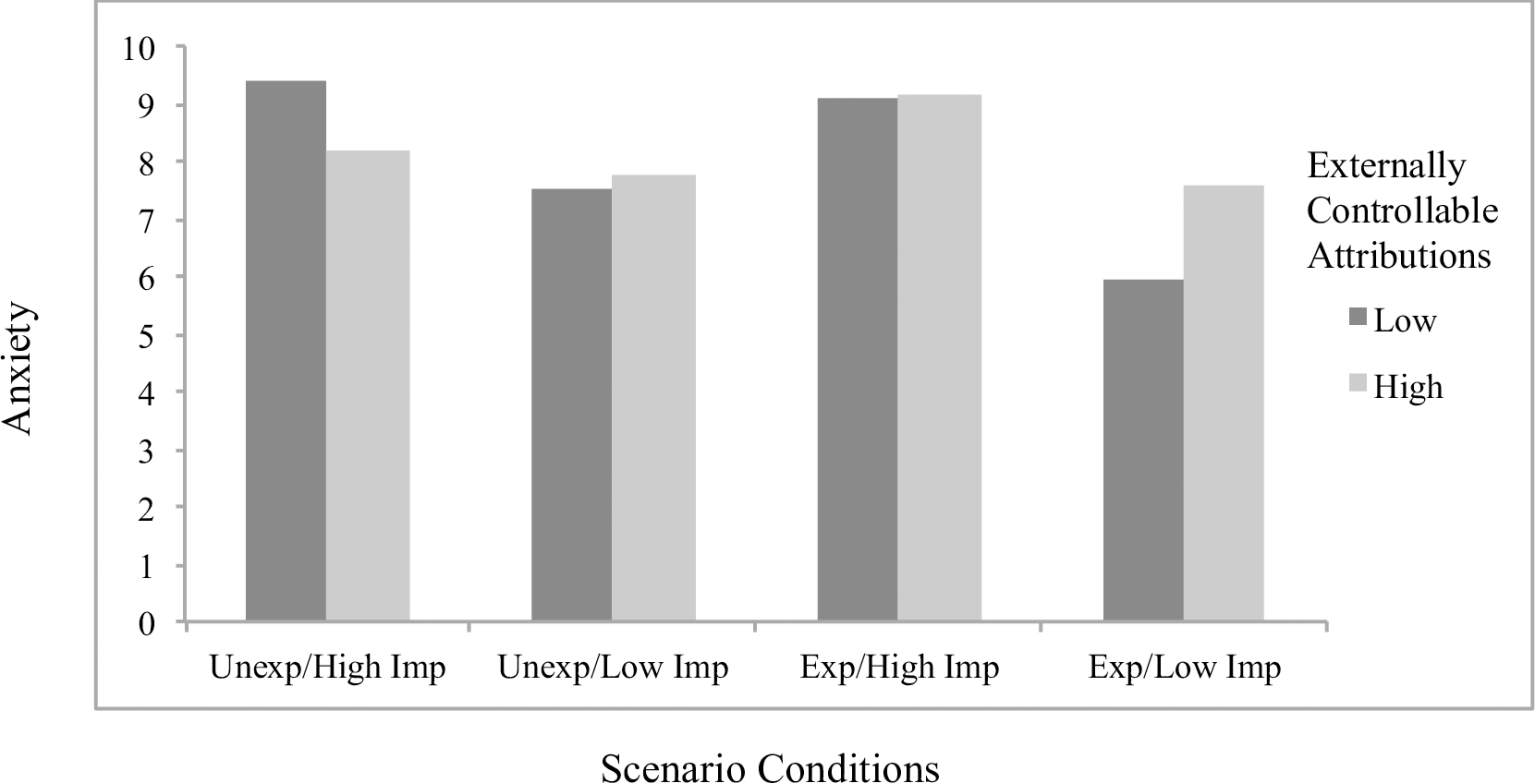
Effects of externally controllable attributions by scenario conditions on anxiety for traditional students.

**Fig 4:**
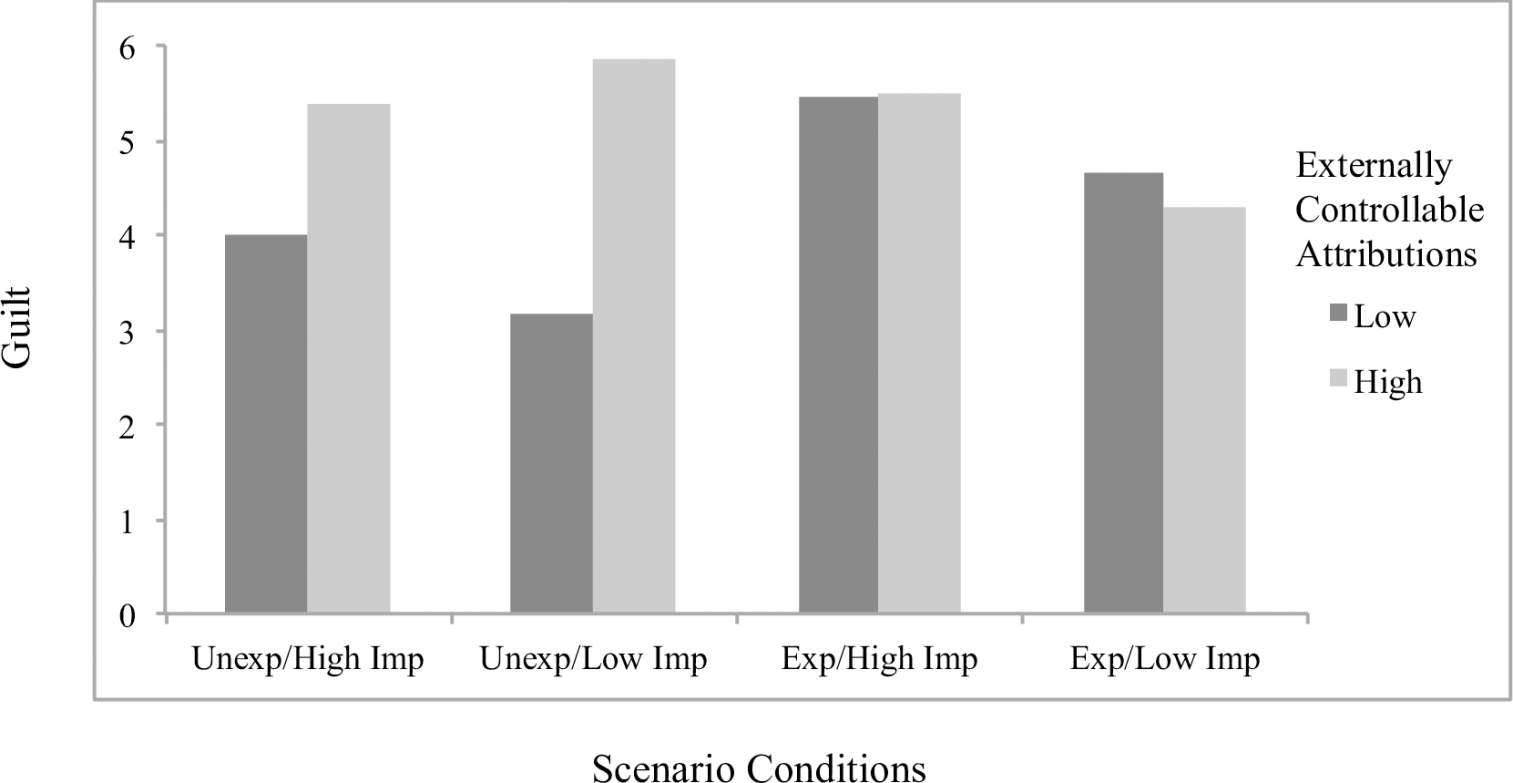
Effects of externally controllable attributions by scenario conditions on guilt for traditional students.

Concerning significant interaction effects, results showed the effects of attributions to stable factors on helplessness (*B* = .23, *p* = .048; Fig 1) and boredom (*B* = .32, *p* = .001; Fig 2) to differ by scenario condition, with significant simple slopes analyses showing stable attributions to predict greater helplessness (*B* = .19, *p* = .022) and boredom (*B* = .27, *p* < .001) specifically for students in the *unexpected/low importance* condition. The effects of externally controllable attributions on anxiety also differed as a function of scenario type (*B* = -.22, *p* = .019; Fig 3), with significant simple slopes showing more anxiety from external attributions in the *expected/low importance* condition (*B* = .12, *p* = .053) and a beneficial effect on anxiety in the *unexpected/high importance* condition that approached significance (*B* = -.092, p = .17). An additional interaction effect further showed the effects of externally controllable attributions on guilt to be moderated by scenario type (*B* = .23, *p* = .045; Fig 4) with simple slopes showing external attributions to predict more guilt only in the *unexpected/low importance* condition (*B* = .20, *p* = .014). All figures show high and low attributions at 1 *SD* above and below the mean.

**Online students:** No significant main or interaction effects of attributions on emotions were found for the online student sample (see Table 5 for effects of internal and personally controllable attributions; see Table 6 for effects of stable and externally controllable attributions).

**Table 5:** Study 1: Hierarchical regression results for internal and personally controllable attributions made by online students.

Predictor	Hope	Guilt	Helplessness	Pride	Shame	Enjoyment	Anxiety	Boredom
Internal attributions								
Step 1/2								
Gender	.25/.27	.65/.52	-.83/-.79	.18/.20	.47/.43	.10/.10	-1.33*/-1.33*	.17/.14
Age	.00/.00	-.03/-.03	-.10***/-.10***	.01/.01	-.03/-.04	.01/.01	-.05*/-.05*	-.03/-.03
Experience	.94***/.95***	-.01/-.03	-.62*/-.59*	-.03/-.03	.09/.08	-.05/-.05	-.20/-.21	-.23/-.25
Attributions	.01/-.02	.04/.16	-.02/-.06	.01/.00	.04/.10	-.01/-.02	.01/.04	.00/.04
U/H	-.90/-.90	.01/-.06	1.19/1.15	-.04/-.04	.03/.00	-.13/-.12	1.86**/1.91**	-.03/.02
U/L	-.29/-.33	-1.12/-1.02	1.05/1.03	-.23/-.23	.13/.22	.10/.07	.30/.40	-.07/.00
E/H	-1.60**/-1.61**	-.38/-.32	-.28/-.25	.46/.47	-.38/-.34	.45/.43	1.52*/1.53*	-.53/-.54
*R*^2^	.11**/.12	.04/.08	.14***/.16	.05/.06	.03/.04	.03/.05	.14***/.15	.03/.05
Attributions X U/H	/.05	/-.27	/.01	/.01	/-.10	/.03	/.02	/.01
Attributions X U/L	/.07	/-.19	/.04	/.00	/-.14	/.04	/-.12	/-.09
Attributions X E/H	/.02	/-.08	/.14	/.04	/-.02	/-.04	/-.04	/-.08
Personally controllable attributions								
Step 1/2								
Gender	.17/.15	.62/.64	-.70/-.72	.19/.20	.51/.53	.11/.09	-1.26*/-1.24*	.13/.10
Age	.00/.01	-.03/-.03	-.10***/-.10***	.01/.01	-.04/-.04	.01/.01	-.05*/-.05*	-.03/-.02
Experience	.91***/.89***	-.07/-.06	-.55/-.55	-.03/-.04	.07/.09	-.04/-.04	-.17/-.19	-.25/-.29
Attributions	.04/.08	.03/.02	-.03/-.05	.00/.00	.02/.05	-.01/-.01	-.01/-.01	.02/.03
U/H	-.85/-.83	-.06/-.07	1.36*/1.36*	-.04/-.04	-.02/-.04	-.13/-.13	1.95***/1.97***	-.02/.02
U/L	-.19/-.20	-1.16/-1.16	1.16/1.17	-.23/-.23	.12/.13	.07/.08	.42/.39	-.07/-.08
E/H	-1.62**/-1.55**	-.49/-.52	-.07/-.06	.46/.44	-.45/-.46	.46/.49	1.64**/1.64**	-.55/-.47
*R*^2^	.13**/.14	.04/.04	.16***/.16	.05/.05	.03/.03	.03/.04	.15***/.15	.03/.07
Attributions X U/H	/-.01	/.00	/.03	/.01	/-.07	/.01	/.02	/.06
Attributions X U/L	/-.08	/.02	/.05	/-.01	/-.02	/.01	/-.06	/-.06
Attributions X E/H	/-.08	/.03	/.01	/.01	/-.01	/-.02	/.00	/-.06

*Note*. Experience reflects computer experience compared to others. Attributions refer to attributions for relevant section. U/H = unexpected/high importance, U/L = unexpected/low importance, and E/H = expected/high importance conditions. Unstandardized *B* coefficients and *R*^2^ values are provided for regressions on study measures. Significance of *R*^2^ values indicates two-tailed significance of change from previous step. For gender: 0 = females, 1 = males.

*p ≤ .05.

**p ≤ .01.

***p ≤ .001

**Table 6:** Study 1: Hierarchical regression results for stable and externally controllable attributions made by online students.

Predictor	Hope	Guilt	Helplessness	Pride	Shame	Enjoyment	Anxiety	Boredom
Stable attributions								
Step 1/2								
Gender	.28/.38	.65/.70	-.80/-.79	.19/.25	.49/.51	.09/.12	-1.27*/-1.27*	.18/.29
Age	.00/.00	-.03/-.04	-.10***/-.10***	.01/.01	-.04/-.04	.01/.01	-.05*/-.05*	-.03/-.03
Experience	.96***/.96***	-.03/-.09	-.60*/-.61*	-.03/-.04	.10/.06	-.05/-.05	-.19/-.22	-.23/-.24
Attributions	-.05/-.11	.08/.24	.08/.07	.01/-.02	.13/.26	.01/-.03	-.03/.08	-.03/-.05
U/H	-.84/-.79	-.16/-.21	1.31*/1.32*	-.05/-.03	-.18/-.25	-.13/-.11	1.99***/1.95***	-.01/.03
U/L	-.16/-.22	-1.31/-1.36	1.06/1.14	-.24/-.23	-.11/-.19	.07/.05	.48/.40	-.05/-.11
E/H	-1.61**/-1.56**	-.43/-.47	-.05/.06	.46/.56	-.39/-.50	.46/.48	1.62**/1.50*	-.55/-.50
*R*^2^	.13**/.14	.05/.08	.17***/.18	.05/.08	.07/.09	.03/.04	.15***/.16	.03/.05
Attributions X U/H	/.03	/-.25	/.01	/.01	/-.20	/.03	/-.17	/-.02
Attributions X U/L	/.17	/-.20	/-.06	/.03	/-.14	/.07	/-.10	/.12
Attributions X E/H	/.08	/-.11	/.12	/.10	/-.15	/.04	/-.16	/.06
Externally controllable attributions								
Step 1/2								
Gender	.42/.49	.37/.27	-1.11/-1.11	.24/.25	.72/.67	.13/.14	-1.36*/-1.37*	.20/.20
Age	.01/.01	-.04/-.05	-.12***/-.12***	.01/.01	-.04/-.04	.01/.01	-.06*/-.06*	-.03/-.03
Experience	.91***/.91***	-.07/-.13	-.57*/-.55	-.03/-.03	.15/.15	-.05/-.05	-.16/-.16	-.30/-.34
Attributions	-.01/.01	.03/-.07	.04/.07	01/.01	.01/-.01	.00/.01	.01/.01	.05/.00
U/H	-.76/-.77	.00/.05	1.37*/1.36*	-.06/-.06	-.17/-.15	-.13/-.13	2.03***/2.03***	.10/.12
U/L	-.14/-.16	-1.06/-1.06	1.25/1.24	-.25/-.26	.18/.18	.08/.07	.62/.62	.01/-.01
E/H	-1.44*/-1.41*	-.43/-.47	-.29/-.37	.48/.47	-.38/-.43	.47/.47	1.59*/1.59*	-.44/-.42
*R*^2^	.10*/.12	.03/.08*	.20***/.21	.06/.06	.03/.04	.03/.04	.16***/.16	.06/.07
Attributions X U/H	/-.08	/.22	/.01	/.00	/.08	/-.01	/.02	/.07
Attributions X U/L	/.01	/.12	/-.03	/.01	/.02	/.00	/.01	/.09
Attributions X E/H	/.02	/.02	/-.10	/-.01	/-.05	/-.01	/.02	/.05

*Note*. Experience reflects computer experience compared to others. Attributions refer to attributions for relevant section. U/H = unexpected/high importance, U/L = unexpected/low importance, and E/H = expected/high importance conditions. Unstandardized *B* coefficients and *R*^2^ values are provided for regressions on study measures. Significance of *R*^2^ values indicates two-tailed significance of change from previous step. For gender: 0 = females, 1 = males.

*p ≤ .05.

**p ≤ .01.

***p ≤ .001

### Study 1 discussion

#### Study hypotheses

Consistent with Weiner’s [21] attribution theory, study findings align with Hypothesis 1 in that assuming personal responsibility for technology problems, by way of attributions to internal factors, predicted lower levels of computing-related helplessness for traditional students. Conversely, attributions to external factors under the control of others predicted higher levels of negative emotions concerning academic computing for traditional students, specifically following moderately serious (unexpected, low importance) or non-serious (expected, low importance) computing problems. As such, these results also partially support Hypothesis 4 in which the weakest effects of causal attributions were expected in the least serious computing condition (expected, low importance). However, a marginally significant post-hoc contrast showed externally controllable attributions to also predict *better* levels of anxiety specifically following serious computing failures (unexpected, high importance), thus directly contradicting both Hypotheses 1 and 4.

Concerning the anticipated effects of personally controllable attributions, Hypothesis 2 was partially supported with these attributions found to significantly predict lower levels of computing-related helplessness, specifically for traditional students. However, the relatively weaker magnitude and lack of other emotional benefits of personally controllable attributions following failure experiences does not support Hypothesis 2 and is not consistent with previous findings concerning the emotional and achievement benefits of students’ personally controllable attributions for poor academic performance [49–50]. This lack of empirical support for Hypothesis 2 thus suggests that the benefits of controllable attributions may not generalize across academic domains (e.g., computing vs. exam performance).

According to Hypothesis 3, students’ attributions for technology-related problems to factors that were stable over time were expected to predict less positive and more negative emotions concerning academic computing. Our findings support this hypothesis in that stable attributions were found to predict greater helplessness and boredom, specifically among traditional students. Additionally, these findings partially support Hypothesis 4 in showing these effects specifically following more serious computing failures, with detrimental effects of stable attributions on helplessness and boredom observed specifically for participants in response to the *unexpected/low importance* failure scenario. These findings for stable attributions are directly consistent with Weiner’s [21] attribution theory as well as previous research on attributions for poor academic performance [51] highlighting the detrimental nature of stable attributions for coping with failure in academic settings.

As outlined above, the study findings also provided mixed results concerning Hypothesis 4, which proposed that the emotional benefits and risks of causal attributions would be most evident following the most serious computing failure scenario (*unexpected*, *high importance*), and conversely, least evident in the least serious condition (*expected*, *low importance*). Consistent with this hypothesis, the effects of causal attributions on students’ computing-related emotions were largely non-significant in the least serious condition. Contrary to this hypothesis, however, were findings showing emotional disadvantages of stable and externally controllable attributions primarily with respect to a *moderately serious* computing failure (as opposed to the most serious) and externally controllable attributions to predict greater anxiety in *only* the least serious condition. Moreover, externally controllable attributions were found to predict *lower* (not higher) anxiety levels in the most serious computing failure condition (marginally significant simple slope)–a finding also directly contradicting Hypothesis 4.

As such, the interaction findings from Study 1 provide novel results suggesting that the emotional disadvantages of attributions for academic computing problems to stable factors, and factors under the control of others, can be anticipated following not only the most serious computing failures but also less serious failure experiences that are simply unexpected. Although this general lack of a combined impact of importance and expectedness is not directly aligned with Weiner’s [21] theory, or expectancy-value theories more generally (e.g., [52]), this result is nonetheless consistent with scenario research showing unexpectedness of academic failure experiences to elicit greater causal search than importance (or the combination of importance and expectedness [31,53]).

Findings contrary to study hypotheses also showed externally controllable attributions to predict *greater* anxiety in the *least* serious condition, as well as *lower* anxiety in the *most* serious condition. These findings are opposite of those typically expected when applying Weiner’s [21] theory in academic settings, and instead suggest that external attributions may be self-protective following academic computing failures (e.g., [54–55]). The unexpected finding of externally controllable attributions predicting guilt also supports the notion that such attributions may promote “activating emotions” that can lead to academic engagement [22]. However, taken together with a general lack of effects for attributions in the most serious failure condition (*unexpected*, *high importance*), it is also possible that the notably emotion-eliciting nature of this scenario may have reduced significant results due to ceiling effects (see Scenario Condition Effects section). Finally, the lack of significant findings for online students, perhaps due to differences in the relative strength of covariates as compared to the traditional sample (e.g., computing experience), further suggests that these students may respond differently than more traditional students to academic computing problems, irrespective of the type of computer problem they experience.

#### Limitations and open questions

With respect to limitations of our scenario findings, it is possible that the hypothetical nature of the computing failure scenarios may not have elicited sufficient causal search (e.g., relative to actual failure events) thereby precluding significant effects. Thus, although causal attributions have been previously assessed using hypothetical scenarios in relation to causal search [31] and emotions [56], more ecologically valid experiential methods may be needed to replicate the findings observed (e.g., manipulated technology failures of varying severity). The scenario study is also limited in employing measures of causal attributions and emotions that assessed students’ overall perceptions of academic computing problems as opposed to evaluating students’ attributions and emotions concerning a specific academic computing event. Whereas the present methodology is consistent with prior research on the importance of assessing students’ emotions in a domain-specific manner (e.g., academic computing vs. academics more generally [57–59]), the findings from Study 1 should be interpreted in light of a lack of situational specificity that would otherwise be afforded by task-specific measures (e.g., word processing vs. online research problems) or qualitative inquiry concerning specific computing experiences (e.g., open-ended writing, in-depth interviews). Finally, as significant findings were observed only for traditional students, this suggests that students enrolled in online post-secondary programs may have perceived the hypothetical scenarios differently than traditional students and that alternate experimental methods may be more effective for observing effects in this population. To address the need for more intensive experimental methods to replicate the scenario findings, Study 2 evaluated the effects of students’ causal attributions on their emotional responses to actual, manipulated technology failures of varying severity.

## Study 2: Experimental method

### Methods

#### Traditional students

The final traditional student sample consisted of 100 undergraduates enrolled at a research-intensive North American university who were recruited in February and March of 2014. Participants’ mean age was 20.48 years (*SD* = 2.51), 72.00% were female, the average self-reported final high school grade was 88.17% (*SD* = 6.41), and 60.00% spoke English as a first language. Participants’ year of study varied (Year 1/2/3/4/5+: 36.00%/28.00%/20.00%/13.00%/3.00%), as did their faculty affiliations (arts: 37.00%, education: 24.00%, science: 23.00%, other: 16.00%). The aforementioned sample consisted only of cases without missing data following the experimental manipulation, as a Little’s MCAR test revealed no patterns of missing responses, Χ^2^(90, *N* = 182) = 90.60, *p* = .46.

#### Online students

The final online student sample consisted of 137 undergraduates recruited from a large, online North American university who were recruited between April and July of 2014. Participants’ mean age was 32.50 years (*SD* = 9.79), 78.10% were female, the average self-reported final high school grade was 81.22% (*SD* = 8.85), and 89.80% spoke English as a first language. The participant sample was varied with respect to program year (Year 1/2/3/4/5+: 35.80%/14.60%/21.90%/10.20%/16.80%) and faculty affiliation (humanities/social sciences: 46.00%, health disciplines: 21.20%, science/technology: 9.50%, business: 7.30%, other: 16.00%). A Little’s MCAR test did not reveal any patterns in missing data, Χ^2^(128, *N* = 203) = 106.86, *p* = .91, therefore the final study sample excluded participants with missing responses following the experimental manipulation.

#### Procedures

As in Study 1, participants were recruited by email (both samples) and in person (traditional students) to complete a 30 min online study related to academic computing, with participants entered into a draw for one of two cash prizes ($250 each). Prior to completing the demographics items on the first questionnaire page, participants were presented with a preamble suggesting that problems with the survey website were either expected or not expected (expectedness manipulation). Following the subsequent attribution measures, participants were presented a segment of a recent *New York Times* article on medical advancements related to the collection of whale DNA and asked to write a summary of the segment that was either one or two paragraphs in length, in which they summarize and discuss the article (importance manipulation). Immediately following the summary page, participants were presented with a manipulated error message indicating that an error had occurred and that the summary text they had entered was lost.

As such, the preamble content (expected vs. unexpected) combined with the extent of data lost (low vs. high importance) comprised the four experimental conditions outlined below. Participants were randomly assigned to one of the four conditions at the beginning of the study, and the emotion measures were evaluated after the simulated loss of text. At the conclusion of the study, participants were provided debriefing information regarding the study protocols. The experimental conditions were developed based on findings showing time lost due to computer errors to have strong relationships with computer anxiety, self-efficacy, and mood [60], with error messages identified as the most frequently experienced cause of computer-related frustration in students [61].

1. Expected/High Importance. The survey preamble highlighted possible problems with the survey website as follows: “Thank you for your participation in this study. Please note that some participants have experienced technical difficulties with the study website. If you encounter any problems while completing the study, please let us know on the comments page at the end of the study.” The requested length of the summary of the article segment was two paragraphs (approximately 10 sentences; Traditional/Online *n*s = 27/40).
2. Expected/Low Importance. Participants were provided the same preamble forecasting potential survey problems as in the preceding condition, however the requested length of summary of the article content was only one paragraph (approximately five sentences; Traditional/Online *n*s = 24/30).
3. Unexpected/High Importance. Participants were provided the following typical preamble prior to the survey in which potential survey problems were not addressed: “Thank you for your participation in this study. Please feel free to provide any feedback on the comments page at the end of the study.” As in the initial study condition, students were requested to write two paragraphs summarizing the article content (Traditional/Online *n*s = 28/31).
4. Unexpected/Low Importance. Participants were provided the same nondescript preamble as in the preceding condition, however the requested length of the article summary was only one paragraph (Traditional/Online *n*s = 21/36).

#### Study measures

Descriptive statistics for the self-report study measures (means, standard deviations, ranges, internal reliability) for both the traditional and online samples are provided in Table 7.

**Table 7:** Study 2: Descriptive statistics.

	*Traditional Students*				*Online Students*			
	*M*	*SD*	Range	α	*M*	*SD*	Range	α
*Covariates*								
Age	20.48	2.51	17–39	-	32.50	9.79	19–68	-
Computer experience	3.71	0.80	2–5	-	3.93	0.77	2–5	-
*Attributions*								
Locus of causality	10.40	6.01	3–27	.78	10.09	5.76	3–27	.76
Personal control	15.17	7.32	3–27	.85	15.07	7.86	3–27	.90
Stability	10.44	5.14	3–27	.47	10.14	5.00	3–27	.34
External control	12.37	6.68	3–27	.78	13.85	7.17	3–27	.84
*Emotions*								
Hope	4.37	2.60	1–9	-	4.93	2.60	1–10	-
Guilt	3.09	2.48	1–8	-	1.99	1.64	1–8	-
Helplessness	4.46	2.67	1–10	-	3.96	2.72	1–10	-
Pride	3.19	2.64	1–10	-	3.88	2.94	1–10	-
Shame	2.43	2.12	1–8	-	1.87	1.72	1–9	-
Enjoyment	3.00	2.71	1–10	-	3.54	3.12	1–10	-
Anxiety	4.44	3.11	1–10	-	3.49	2.90	1–10	-
Boredom	3.08	2.44	1–10	-	3.03	2.32	1–9	-

**Causal attributions:** Causal attributions were assessed using the same modified version of the Revised Causal Dimension Scale (CDSII) [43] used in Study 1 that consisted of twelve 9-point items measuring perceived locus of causality, personal control, stability, and external control. As in Study 1, the internal reliability for the stability measure was low (α = .41) but nonetheless retained given similar reliability levels found in prior research [43–44].

**Outcome and activity emotions:** Similar to Study 1, eight 10-point items (1 = *not at all*, 10 = *very much so*) assessed students’ emotions “when experiencing problems using computers for school (including tablets, smartphones, etc.)” following from existing measures of emotions concerning academic performance [25,29]. The items evaluated both outcome-related emotions proposed in Weiner’s [21] attribution theory to follow directly from causal attributions for specific events (hope, guilt, helplessness, pride, shame), as well as emotions outlined in Pekrun’s [22] control-value theory to occur primarily during activities prior to a specific outcome (enjoyment, anxiety, boredom).

**Computing experience:** As in Study 1, self-rated computing experience in relation to others was assessed using a single 5-point item (1 = *none*, 5 = *excellent*) and included as a covariate in our main analyses below.

### Results

#### Preliminary analyses

**Participant attrition:** Given an initial sample size of *N* = 182, it should be noted that the final *N* = 100 reflected substantial attrition within the study session, with 45% of participants missing responses following the experimental manipulation. Follow-up analyses further showed significant differences in attributions to stable factors between participants who completed the study and those who did not, *t*(146) = 1.96, *p* = .052, *d* = .35, with those who completed the study reporting more stable attributions (*M* = 10.44, *SD* = 5.14) than those who did not (*M* = 8.79, *SD* = 4.35). Within-session attrition was similarly observed following the experimental manipulation for online students, reducing the sample size by 33% (initial *N* = 203). After comparing the final participant sample with students who quit the study following the experimental manipulation, no significant differences in age, gender, program year, or high school grades were found for either traditional or online students. Additionally, no significant differences were observed on the attribution measures for online students as a function of study attrition.

**Initial differences:** Independent-samples *t*-tests exploring possible initial differences on study variables due to gender and sample type were conducted. In the traditional student sample, females reported higher attributions to internal factors (*M* = 11.17, *SD* = 6.19; *t*(96) = 2.10, *p* = .039, *d* = .49) compared to males (*M* = 8.37, *SD* = 5.07). Females also reported higher levels of helplessness (*M* = 5.09, *SD* = 2.67; *t*(66.48) = 4.35, *p* < .001, *d* = .91), shame (*M* = 2.81, *SD* = 2.33; *t*(96) = 4.11, *p* < .001, *d* = .76), and anxiety (*M* = 5.04, *SD* = 3.18; *t*(61.83) = 3.71, *p* < .001, *d* = .79) compared to males (*M* = 2.93, *SD* = 2.00; *M* = 1.46, *SD* = .92; *M* = 2.85, *SD* = 2.31; respectively). For online students, females similarly reported greater helplessness (*M* = 4.36, *SD* = 2.77; *t*(57.81) = 3.75, *p* < .001, *d* = .72), guilt (*M* = 2.15, *SD* = 1.77; *t*(95.33) = 3.09, *p* = .003, *d* = .52), and anxiety (*M* = 3.79, *SD* = 2.97; *t*(51.02) = 2.44, *p* = .018, *d* = .49) relative to their male counterparts (*M* = 2.61, *SD* = 2.01; *M* = 1.43, *SD* = .84; *M* = 2.46, *SD* = 2.43; respectively).

When assessed across experimental conditions, initial differences in emotions were found between traditional and online students, with traditional students reporting significantly greater feelings of guilt (*M* = 3.09, *SD* = 2.48; *t*(157.41) = 3.81, *p* < .001, *d* = .52), shame (*M* = 2.43, *SD* = 2.12; *t*(182.40) = 2.14, *p* = .034, *d* = .29), and anxiety (*M* = 4.44, *SD* = 3.11; *t*(226) = 2.37, *p* = .019, *d* = .32) compared to online students (*M* = 1.99, *SD* = 1.64; *M* = 1.87, *SD* = 1.72; *M* = 3.49, *SD* = 2.90, respectively). Similar to Study 1, the average age of online students was significantly higher (*M* = 32.50, *SD* = 9.79; *t*(159.89) = -13.77, *p* < .001, *d* = 1.68) than that of traditional students (*M* = 20.48, *SD* = 2.51). Additionally, online students reported having significantly more computing experience (*M* = 3.93, *SD* = .77; *t*(231) = -2.18, *p* = .030, *d* = .28) than traditional students (*M* = 3.71, *SD* = .80). As in Study 1, traditional and online students were examined separately in the main analyses given significant initial sample differences.

Concerning initial differences between experimental conditions, a MANOVA revealed significant differences in attributions for traditional students, *F*(12, 235.76) = 1.77, *p* = .053, η_p_^2^ = .073, showing students in the *expected/high importance* condition (*M* = 13.24, *SE* = 1.17) to report higher internal attributions compared to students in the *unexpected/high importance* (*M* = 9.25, *SE* = 1.11), *unexpected/low importance* (*M* = 8.90, *SE* = 1.31), and *expected/low importance* conditions (*M* = 10.30, *SE* = 1.22; *F*(3, 92) = 2.75, *p* = .047, η_p_^2^ = .082). Traditional students in the *unexpected/low importance* condition also reported having more computing experience (*M* = 4.19, *SE* = .17) than students in the *unexpected/high importance* (*M* = 3.48, *SE* = .15), *expected/high importance* (*M* = 3.78, *SE* = .15), and *expected/low importance* conditions (*M* = 3.46, *SE* = .16; *F*(3, 95) = 4.58, *p* = .005, η_p_^2^ = .13). ANOVAs showed no initial differences between experimental conditions for attributions, age, or computing experience for online students, or age for traditional students, with Chi-square tests showing no differences in gender proportions between conditions for either sample.

**Correlational analyses:** Correlations between continuous study variables for both the traditional and online samples are presented in Table 8. As in Study 1, strong correlations were observed between internal and personally controllable attributions, with both variables also correlating negatively with attributions to externally controlled factors in each student sample. Positive emotions were once again positively intercorrelated in each sample (e.g., pride and enjoyment), as were negative emotions (e.g., anxiety and helplessness), with negative correlations consistently observed between positive and negative emotions (e.g., pride and anxiety). Similar to Study 1, unanticipated correlations were found for feelings of computing-related boredom, with boredom correlating positively, albeit weakly, with the positive emotions of both hope and pride (*r*s ≤ .21). As correlations for online students showed computing experience to correspond with lower negative emotions (helplessness, guilt, anxiety), more external attributions, and greater enjoyment, with age found to correlate negatively with boredom in this sample, these background measures were included as covariates in the main analyses.

**Table 8:** Study 2: Zero-order correlations.

	1	2	3	4	5	6	7	8	9	10	11	12	13	14
1. Internal attributions	-	.63***	.04	-.29***	.20*	.00	.07	.32***	.06	.24**	.09	-.05	-.04	.10
2. Personally controllable attributions	.64***	-	-.09	-.34***	.16	-.08	.01	.17*	.00	.14	.04	-.14	-.03	.05
3. Stable attributions	-.01	-.17	-	.00	.12	-.02	-.02	.12	-.06	-.01	-.01	-.02	-.02	.07
4. Externally controllable attributions	-.19	-.23*	-.14	-	-.02	-.05	-.12	-.05	.01	.07	-.13	.01	.15	.18*
5. Hope	.03	.05	.05	-.12	-	-.03	-.07	.54***	-.01	.51***	-.02	.15	.07	.10
6. Guilt	.37***	.23*	.15	-.17	.00	-	.25**	.18*	.38***	.15	.12	.19*	-.02	-.18*
7. Helplessness	.15	.02	.10	.00	-.25**	.21*	-	-.17	.38****	-.31***	.67***	.22**	-.03	-.37***
8. Pride	.11	-.02	.11	-.09	.59***	.17	-.30**	-	.16	.70***	-.13	.17	.10	.14
9. Shame	.46***	.25*	.28**	.00	-.06	.57***	.40***	-.02	-	.06	.40***	.35***	-.09	-.06
10. Enjoyment	.01	-.09	.13	-.05	.52***	.11	-.39***	.81***	-.07	-	-.32***	.16	-.05	.24**
11. Anxiety	.34***	.19	.14	-.09	-.24*	.36***	.57***	-.41***	.49***	-.44***	-	.09	.01	-.27**
12. Boredom	.29**	.13	.08	.07	.20*	.35***	.06	.21*	.38***	.05	.17	-	-.34***	-.08
Covariates														
13. Age	-.11	-.07	-.04	-.14	.05	-.13	.00	.07	-.11	-.03	-.06	.05	-	-.07
14. Computer experience	.09	.17	.03	-.07	.02	.04	-.14	.18	-.04	.07	-.04	-.11	-.05	-

*Note*. Correlations below the diagonal are for traditional students; correlations above the diagonal are for online students.

*p ≤ .05.

**p ≤ .01.

***p ≤ .001

**Experimental condition effects:** For traditional students, a MANOVA showed students in the *expected/low importance* condition (*M* = 1.82, *SE* = .50) to report less boredom than students in the *expected/high importance* (*M* = 3.64, *SE* = .47), *unexpected/high importance* (*M* = 3.52, *SE* = .45), and *unexpected/low importance* conditions (*M* = 3.00, *SE* = .52; *F*(3, 90) = 2.96, *p* = .037, η_p_^2^ = .090). Similar to Study 1, a marginally significant effect showed traditional students in the *expected/high importance* condition to report greater pride than in the other conditions, *F*(3, 90) = 2.48, *p* = .066, η_p_^2^ = .076. No experimental effects on emotions were found for online students.

#### Rationale for main analyses

The hypothesized main effects (attributions on emotions) and interaction effects (attributions by experimental conditions on emotions) were evaluated using hierarchical linear regressions including gender, age, and computing experience as covariates. In all regression analyses, Step 1 included the covariates and effects of experimental conditions and attributions, with condition by attribution interaction terms included in Step 2. To minimize potential multicollinearity between the attribution dimensions (e.g., internality vs. personal controllability), and to preserve statistical power, the four attribution dimensions were evaluated independently in separate regression analyses. As in Study 1, the experimental condition variables were dummy coded (*expected/low importance* condition as reference group), continuous variables were mean-centered prior to analysis, the traditional and online student samples were assessed separately, and simple slopes analyses were conducted by reverse coding experimental conditions having significant interactions with the reference group.

#### Main analyses

**Fig 5:**
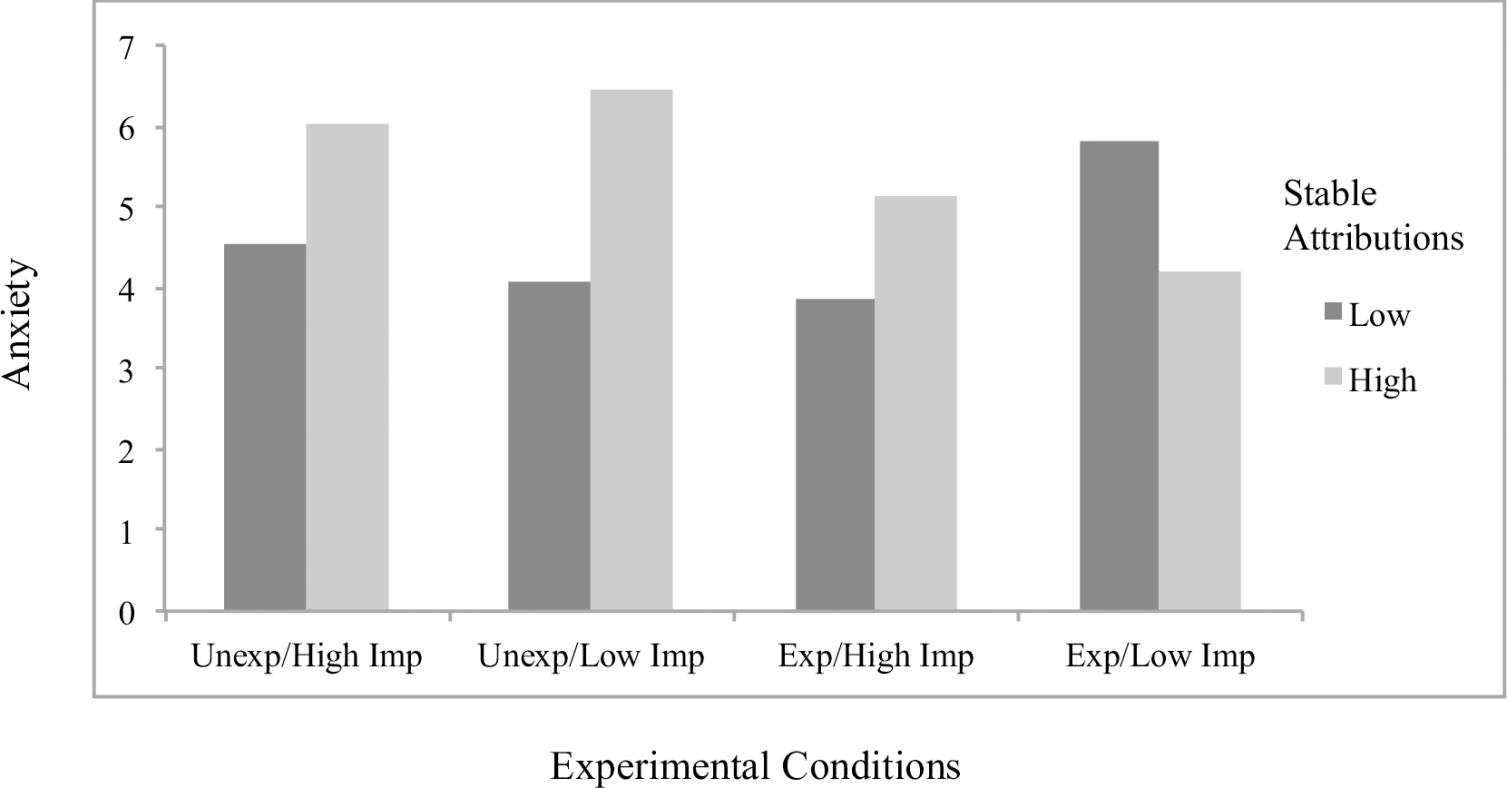
Effects of stable attributions by experimental conditions on anxiety for traditional students.

**Traditional students:** Concerning the main effects of the attribution dimensions, internal attributions for computing difficulties predicted higher levels of guilt (*B* = .15, *p* = .001), shame (*B* = .17, *p* < .001), anxiety (*B* = .18, *p* = .002), and boredom (*B* = .15, *p* = .001). Personally controllable attributions predicted stronger feelings of shame (*B* = .072, *p* = .015) and anxiety (*B* = .089, *p* = .053), and stable attributions predicted stronger feelings of shame (*B* = .10, *p* = .016). When interaction effects were included, only a suppression effect showing internal attributions to predict more shame was significant (*B* = .21, *p* = .005; see Table 9 for effects of internal and personally controllable attributions). A significant interaction effect also showed the effects of stable attributions on anxiety to be moderated by experimental condition (*B* = .39, *p* = .041; Fig 5), with simple slopes showing stable attributions to predict greater anxiety specifically for students in the *unexpected/low importance* condition (*B* = .23, *p* = .059; see Table 10 for effects of stable and externally controllable attributions).

**Table 9:** Study 2: Hierarchical regression results for internal and personally controllable attributions made by traditional students.

Predictor	Hope	Guilt	Helplessness	Pride	Shame	Enjoyment	Anxiety	Boredom
Internal attributions								
Step 1/2								
Gender	.18/.12	.11/-.06	-1.91**/-1.86	.72/.45	-.85*/-.95*	.95/.75	-1.78*/-1.80*	.75/.69
Age	.02/-.01	-.11/-.14	.01/.03	.07/.03	-.06/-.08	-.05/-.08	-.04/-.05	.05/.03
Experience	.21/.15	-.04/-.06	-.09/-.05	.59/.61	-.16/-.16	.08/.11	-.06/-.10	-.69*/-.70*
Attributions	-.01/.08	.15***/.18	.06/-.03	.03/.62	.17***/.21**	.00/.03	.18**/.23	.15***/.09
U/H	.21/.26	.73/.85	.17/.13	1.20/1.31	.30/.33	.49/.54	.32/.34	1.83**/1.91**
U/L	-.68/-.75	.71/.74	-.49/-.44	-.07/.09	.53/.57	.16/.31	.25/.20	1.84*/1.84*
E/H	.90/1.06	.45/.67	-.87/-1.02	1.66*/1.92	.13/.24	1.32/1.51	-.90/-.83	1.94**/2.02**
*R*^2^	.05/.07	.16*/.19	.17*/.18	.14*/.18	.30***/.31	.06/.08	.21**/.21	.23**/.24
Attributions X U/H	/-.03	/.09	/.05	/.09	/-.01	/.02	/-.03	/.13
Attributions X U/L	/-.16	/-.04	/.15	/.07	/-.03	/.05	/-.11	/.03
Attributions X E/H	/-.14	/-.11	/.14	/-.13	/-.10	/-.12	/-.08	/.03
Personally controllable attributions								
Step 1/2								
Gender	.22/.04	-.27/-.42	-2.07***/-1.85	.60/.29	-1.28**/-1.36	.91/.59	-2.18**/-2.17	.38/.10
Age	.02/-.02	-.15/-.19	-.01/.01	.05/-.01	-.10/-.12	-.06/-.11	-.07/-.08	.01/-.01
Experience	.18/.17	.02/.00	-.05/-.03	.65/.62	-.07/-.08	.15/.12	.01/.01	-.62/-.65*
Attributions	.01/.05	.08/.01	.01/-.01	-.02/-.02	.07*/.08	-.04/-.04	.09*/.08	.07/.07
U/H	.27/.46	.96/1.12	.18/.16	1.11/1.31	.47/.50	.31/.46	.53/.60	1.99**/1.94**
U/L	-.64/-.53	.63/.60	-.56/-.66	-.17/-.06	.40/.44	.06/.17	.11/.10	1.73*/1.83*
E/H	.89/1.14	.77/.86	-.76/-.93	1.73/2.01**	.47/.54	1.32/1.58	-.62/-.60	2.24**/2.38***
*R*^2^	.05/.07	.10/.15	.15*/.17	.14/.20	.17*/.17	.08/.13	.15*/.15	.16*/.20
Attributions X U/H	/.01	/.16	/.04	/.07	/-.01	/.04	/.03	/-.03
Attributions X U/L	/-.02	/.16	/-.08	/.12	/.02	/.13	/-.02	/.16
Attributions X E/H	/-.13	/-.01	/.09	/-.13	/-.04	/-.13	/-.01	/-.07

*Note*. Experience reflects computer experience compared to others. Attributions refer to attributions for relevant section. U/H = unexpected/high importance, U/L = unexpected/low importance, and E/H = expected/high importance conditions. Unstandardized *B* coefficients and *R*^2^ values are provided for regressions on study measures. Significance of *R*^2^ values indicates two-tailed significance of change from previous step. For gender: 0 = females, 1 = males.

*p ≤ .05.

**p ≤ .01.

***p ≤ .001

**Table 10:** Study 2: Hierarchical regression results for stable and externally controllable attributions made by traditional students.

Predictor	Hope	Guilt	Helplessness	Pride	Shame	Enjoyment	Anxiety	Boredom
Stable attributions								
Step 1/2								
Gender	.10/.17	-.41/-.36	-2.00/-2.18	.58/.55	-1.34**/-1.34	.67/.73	-2.22/-2.12**	.52/.59
Age	.02/.00	-.16/-.15	-.01/.02	.05/.06	-.11/-.10	-.04/-.04	-.08/-.05	-.01/-.01
Experience	.26/.30	.20/.19	-.04/-.09	.70*/.71	.05/.04	.16/.15	.13/-.03	-.56/-.57
Attributions	.02/.09	.07/-.02	.05/-.04	.04/.00	.10*/.06	.06/.00	.10/-.16	.01/-.01
U/H	.20/.09	.51/.60	.05/.19	1.12/1.16	.01/.06	.41/.47	.03/.27	1.69/1.70
U/L	-.71/-.85	.34/.43	-.64/-.45	-.21/-.19	.14/.18	.09/.14	-.15/.25	1.57/1.60
E/H	.75/.73	.57/.63	-.79/-.81	1.60*/1.57	.25/.26	.90/.93	-.77/-.51	2.43/2.50
*R*^2^	.04/.05	.06/.08	.14/.17	.15*/.16	.16*/.16	.05/.07	.12/.16	.14/.14
Attributions X U/H	/-.04	/.17	/-.03	/.06	/.05	/.15	/.30	/.07
Attributions X U/L	/-.11	/.04	/.17	/.00	/.03	/.01	/.39*	/.02
Attributions X E/H	/-.12	/.13	/.17	/.08	/.06	/.10	/.28	/.00
Externally controllable attributions								
Step 1/2								
Gender	.26/.37	-.25/.00	-2.08***/-2.38***	.65/.71	-1.36/-1.25	.99/1.12	-2.20/-2.23	.27/.31
Age	.01/.02	-.19/-.17	-.02/-.03	.05/.06	-.13/-.12	-.05/-.03	-.11/-.11	-.01/.01
Experience	.17/.07	.08/-.03	-.03/.12	.60/.54	.05/.01	.06/-.03	.10/.04	-.50/-.50
Attributions	-.03/.05	-.07/-.02	-.01/-.10	-.02/.04	.00/-.01	-.02/.04	-.05/.04	.02/.00
U/H	.31/.25	.82/.88	.13/.10	1.24/1.16	.17/.23	.55/.49	.32/.21	1.66/1.64
U/L	-.55/-.49	.70/.74	-.58/-.63	-.05/.04	.24/.23	.23/.39	.10/.07	1.51/1.69
E/H	.88/.83	.74/.94	-.76/-.94	1.72/1.68	.46/.59	1.31/1.40	-.67/-.92	2.24/2.41
*R*^2^	.05/.07	.07/.10	.15*/.19	.14/.16	.11/.12	.06/.09	.12/.13	.12/.14
Attributions X U/H	/-.12	/-.13	/.19	/-.07	/-.04	/-.08	/-.10	/.04
Attributions X U/L	/-.15	/-.08	/.15	/-.15	/.02	/-.18	/-.11	/-.08
Attributions X E/H	/-.06	/.04	/.00	/-.05	/.05	/.00	/-.15	/.08

*Note*. Experience reflects computer experience compared to others. Attributions refer to attributions for relevant section. U/H = unexpected/high importance, U/L = unexpected/low importance, and E/H = expected/high importance conditions. Unstandardized *B* coefficients and *R*^2^ values are provided for regressions on study measures. Significance of *R*^2^ values indicates two-tailed significance of change from previous step. For gender: 0 = females, 1 = males.

*p ≤ .05.

**p ≤ .01.

***p ≤ .001

**Fig 6:**
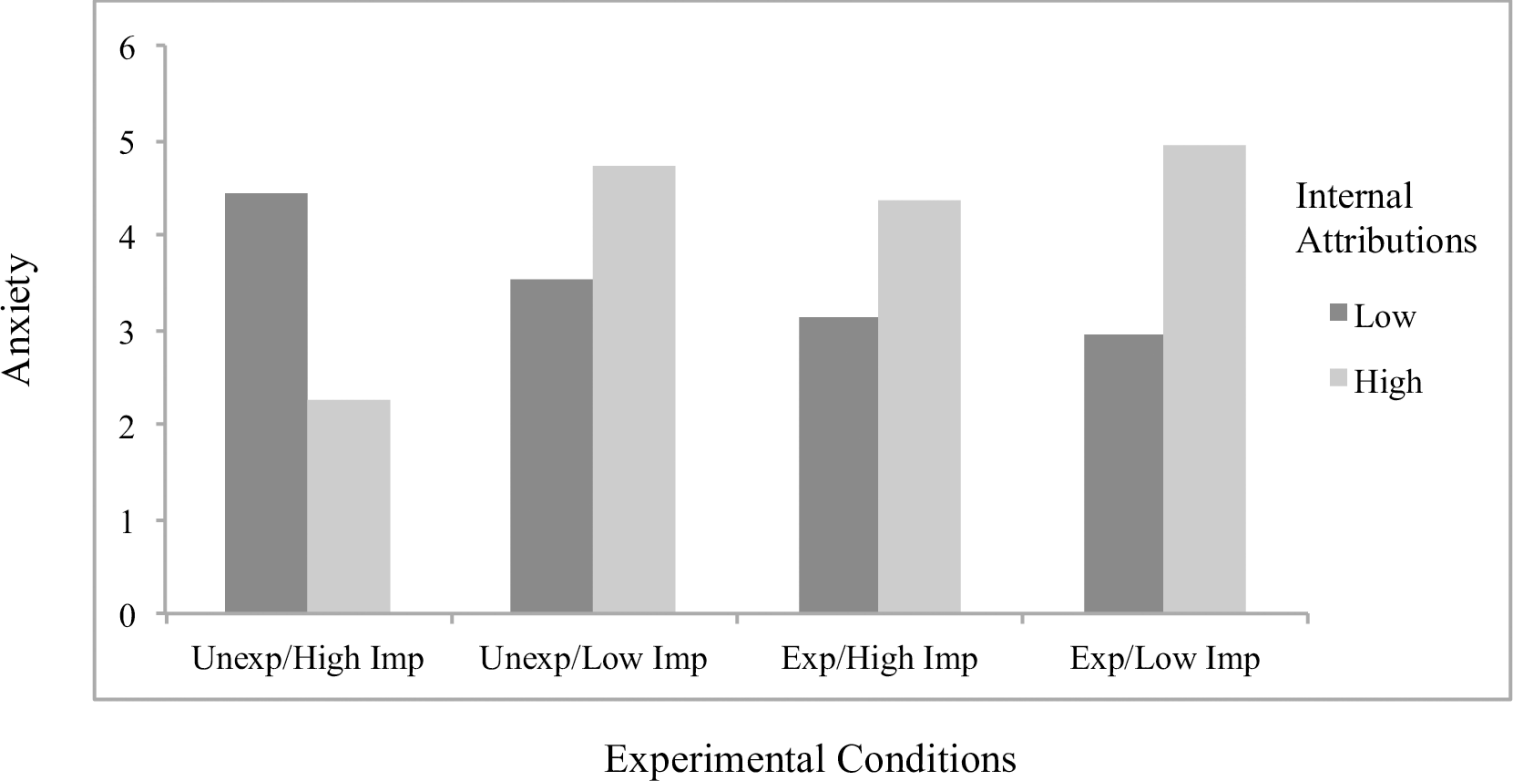
Effects of internal attributions by experimental conditions on anxiety for online students.

**Fig 7:**
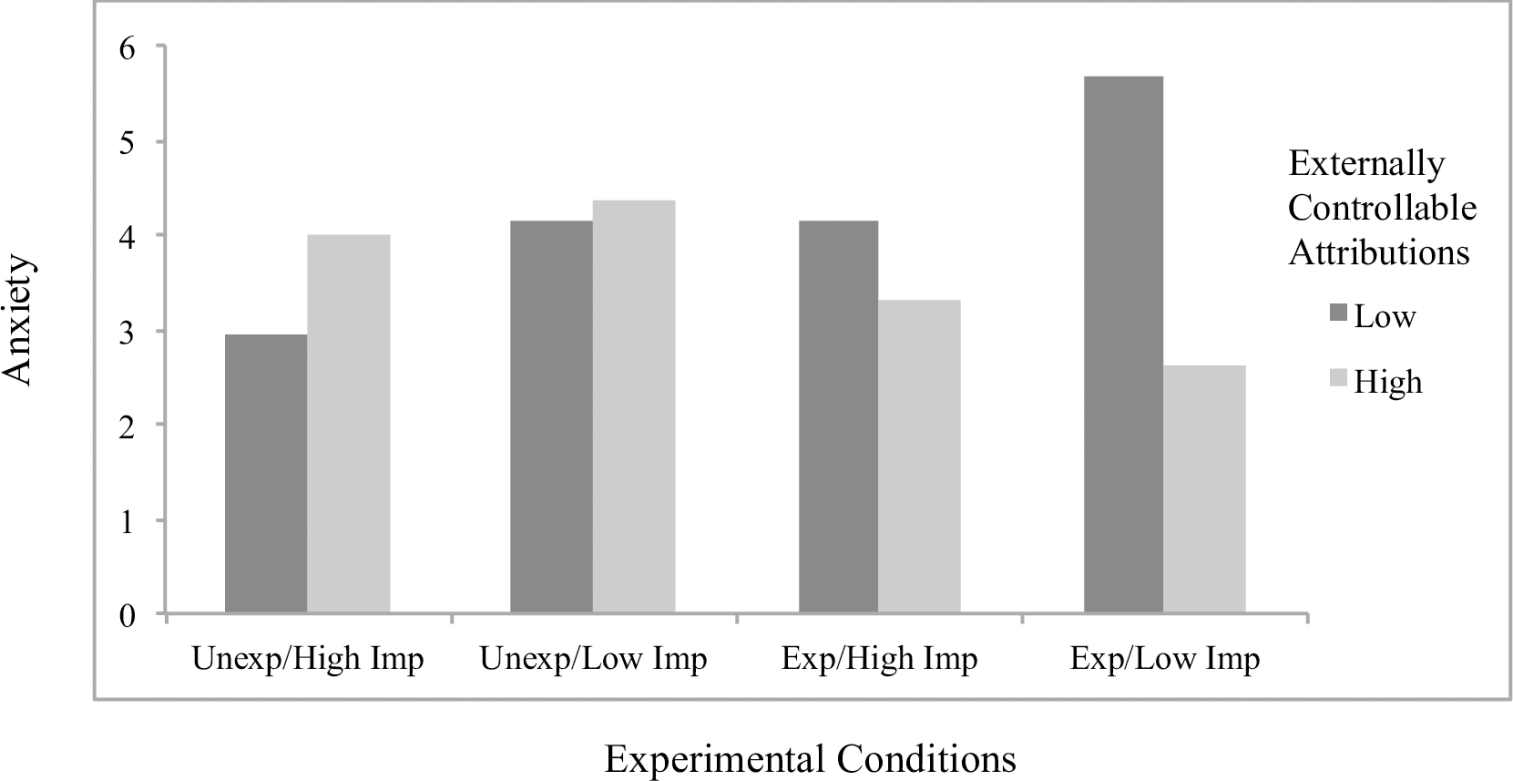
Effects of externally controllable attributions by experimental conditions on anxiety for online students.

**Online students:** Significant main effects of attributions were observed showing internal attributions to predict stronger feelings of pride (*B* = .15, *p* = .001) and enjoyment (*B* = .11, *p* = .028), with significant suppression effects observed in Step 2 showing personally controllable attributions to predict greater hope (*B* = .24, *p* = .001; see Table 11 for effects of internal and personally controllable attributions) and externally controllable attributions to predict lower anxiety (*B* = -.21, *p* = .018; see Table 12 for effects of stable and externally controllable attributions). With respect to interaction effects, results showed the effects of both internal attributions (*B* = -.36, *p* = .018; Fig 6) and externally controllable attributions (*B* = .29, *p* = .016; Fig 7) on anxiety to be markedly different for students in the *unexpected/high importance* condition compared to the reference group. Simple slopes analyses indicated that although internal attributions marginally predicted lower anxiety in the *unexpected/high importance* condition (*B* = -.19, *p* = .097), they also marginally predicted higher anxiety in the *expected/low importance* reference condition (*B* = .17, *p* = .086). Concerning attributions to externally controlled factors, simple slopes showed these attributions to only to predict lower anxiety in the *expected/low importance* condition (*B* = -.21, *p* = .018).

**Table 11:** Study 2: Hierarchical regression results for internal and personally controllable attributions made by online students.

Predictor	Hope	Guilt	Helplessness	Pride	Shame	Enjoyment	Anxiety	Boredom
Internal attributions								
Step 1/2								
Gender	.50/.57	-.47/-.47	-1.35*/-1.26*	-.29/-.27	-.39/-.26	.40/.41	-1.01/-.83	.44/.44
Age	.02/.02	.00/.00	.01/.00	.04/.04	-.02/-.02	-.01/-.01	.01/.01	-.10***/-.10***
Experience	.27/.17	-.34/-.28	-1.11***/-1.17***	.48/.52	-.08/-.13	.79*/.81	-.90/-1.02**	-.23/-.25
Attributions	.09/.19	.00/-.07	.06/.09	.15***/.09	.02/.04	.11*/.09	.07/.17	-.03/.00
U/H	-.12/-.17	-.88/-.88	-.12/-.16	-.81/-.83	-.27/-.33	-1.43/-1.44	-.50/-.60	.23/.23
U/L	-.70/-.69	-.35/-.36	.47/.41	-.94/-.95	-.17/-.22	-.64/-.65	.22/.19	.01/.02
E/H	.04/.09	-.79/-.74	.43/.33	-1.03/-.95	-.32/-.35	-.76/-.74	-.27/-.20	.04/.05
*R*^2^	.07/.10	.09/.11	.18**/.19	.15**/.15	.03/.06	.12*/.12	.11/.16	.16**/.16
Attributions X U/H	/-.26	/.10	/-.11	/.06	/-.15	/.03	/-.36*	/-.05
Attributions X U/L	/-.11	/.08	/.03	/.07	/.03	/.04	/-.07	/-.04
Attributions X E/H	/-.08	/.11	/-.08	/.11	/-.03	/.04	/-.06	/-.03
Personally controllable attributions								
Step 1/2								
Gender	.29/.22	-.48/-.47	-1.32*/-1.30*	-.27/-.28	-.37/-.34	.45/.43	-.94/-.91	.48/.43
Age	.02/.03	.00/.00	.01/.01	.04/.04	-.02/-.01	-.01/-.01	.00/.01	-.10***/-.09***
Experience	.25/.04	-.35/-.32	-1.13***/-1.19***	.46/.33	-.09/-.11	.81/.74	-.93**/-1.04	-.26/-.37
Attributions	.05/.24***	-.01/-.06	.02/.06	.06/.17	.00/.00	.05/.11	.03/.11	-.05/.06
U/H	-.34/-.42	-.75/-.71	-.22/-.20	-.98/-1.02	-.22/-.18	-1.75/-1.78	-.58/-.57	.11/.05
U/L	-.81/-.99	-.25/-.20	.52/.45	-.90/-1.02	-.09/-.11	-.70/-.76	.32/.20	.08/.00
E/H	-.01/-.15	-.69/-.60	.50/.47	-1.23/-1.35	-.27/-.24	-1.05/-1.09	-.15/-.23	-.01/-.12
*R*^2^	.05/.13*	.09/.10	.19***/.20	.08/.10	.03/.04	.10/.11	.11*/.14	.18**/.20
Attributions X U/H	/-.28**	/.05	/-.11	/-.18	/-.05	/-.10	/-.19	/-.13
Attributions X U/L	/-.24**	/.05	/-.02	/-.12	/.03	/-.08	/-.06	/-.13
Attributions X E/H	/-.15	/.07	/-.03	/-.11	/.02	/-.05	/-.08	/-.11

*Note*. Experience reflects computer experience compared to others. Attributions refer to attributions for relevant section. U/H = unexpected/high importance, U/L = unexpected/low importance, and E/H = expected/high importance conditions. Unstandardized *B* coefficients and *R*^2^ values are provided for regressions on study measures. Significance of *R*^2^ values indicates two-tailed significance of change from previous step. For gender: 0 = females, 1 = males.

*p ≤ .05.

**p ≤ .01.

***p ≤ .001

**Table 12:** Study 2: Hierarchical regression results for stable and externally controllable attributions made by online students.

Predictor	Hope	Guilt	Helplessness	Pride	Shame	Enjoyment	Anxiety	Boredom
Stable attributions								
Step 1/2								
Gender	.44/.44	-.49/-.50	-1.38*/-1.36*	-.16/-.15	-.38/-.39	.50/.50	-1.02/-.98	.51/.51
Age	.01/.01	.00/.00	.01/.00	.04/.04	-.02/-.01	-.02/-.01	.01/.00	-.09***/-.09***
Experience	.29/.25	-.36/-.34	-1.12***/-1.15***	.47/.55	-.08/.00	.87*/1.06**	-.91/-1.07	-.24/-.31
Attributions	.05/-.01	.01/.05	.01/-.04	.06/.13	-.02/.09	-.01/.22	.01/-.25	.00/-.06
U/H	-.29/-.25	-.84/-.90	-.10/-.09	-1.02/-.89	-.21/-.20	-1.65/-1.52	-.48/-.44	.26/.23
U/L	-.82/-.78	-.33/-.37	.51/.52	-.92/-.89	-.13/-.15	-.75/-.75	.27/.34	.09/.10
E/H	-.20/-.19	-.74/-.75	.45/.44	-1.45/-1.47	-.30/-.31	-1.19/-1.22	-.27/-.27	.11/.13
*R*^2^	.04/.05	.09/.11	.18**/.18	.08/.09	.03/.04	.09/.13	.10/.13	.15**/.16
Attributions X U/H	/.03	/.00	/.06	/-.20	/-.13	/-.39*	/.27	/.10
Attributions X U/L	/.11	/-.10	/.06	/-.03	/-.12	/-.23	/.32	/.09
Attributions X E/H	/.03	/-.04	/.11	/-.04	/-.11	/-.19	/.35	/-.01
Externally controllable attributions								
Step 1/2								
Gender	.25/.30	-.44/-.45	-1.43*/-1.37*	-.26/-.14	-.37/-.33	.47/.51	-1.09/-.99	.46/.57
Age	.02/.02	.00/.00	.01/.01	.04/.04	-.02/-.02	-.02/-.01	.01.01	-.10***/-.10***
Experience	.31/.28	-.39/-.40	-1.08***/-1.10***	.47/.43	-.10/-.15	.80/.76	-.84*/-.93**	-.31/-.33
Attributions	-.03/-.09	.02/.00	-.03/-.08	.00/-.04	.01/-.09	.02/-.09	-.04/-.21*	.03/.02
U/H	-.30/-.39	-.75/-.76	-.11/-.20	-.90/-.94	-.19/-.34	-1.61/-1.78	-.45/-.68	.18/.17
U/L	-.72/-.83	-.32/-.30	.49/.38	-.84/-.97	-.03/-.14	-.60/-.74	.30/.10	.19/.06
E/H	-.06/-.16	-.68/-.69	.49/.39	-1.27/-1.34	-.27/-.43	-1.08/-1.26	-.16/-.43	.03/.01
*R*^2^	.03/.03	.09/.10	.19***/.19	.06/.07	.03/.07	.09/.10	.11*/.16	.16**/.17
Attributions X U/H	/.09	/.04	/.08	/.11	/.15	/.13	/.29*	/.05
Attributions X U/L	/.05	/.04	/.05	/.00	/.14	/.13	/.23	/-.04
Attributions X E/H	/.07	/.00	/.07	/.03	/.10	/.13	/.15	/.01

*Note*. Experience reflects computer experience compared to others. Attributions refer to attributions for relevant section. U/H = unexpected/high importance, U/L = unexpected/low importance, and E/H = expected/high importance conditions. Unstandardized *B* coefficients and *R*^2^ values are provided for regressions on study measures. Significance of *R*^2^ values indicates two-tailed significance of change from previous step. For gender: 0 = females, 1 = males.

*p ≤ .05.

**p ≤ .01.

***p ≤ .001

**Fig 8:**
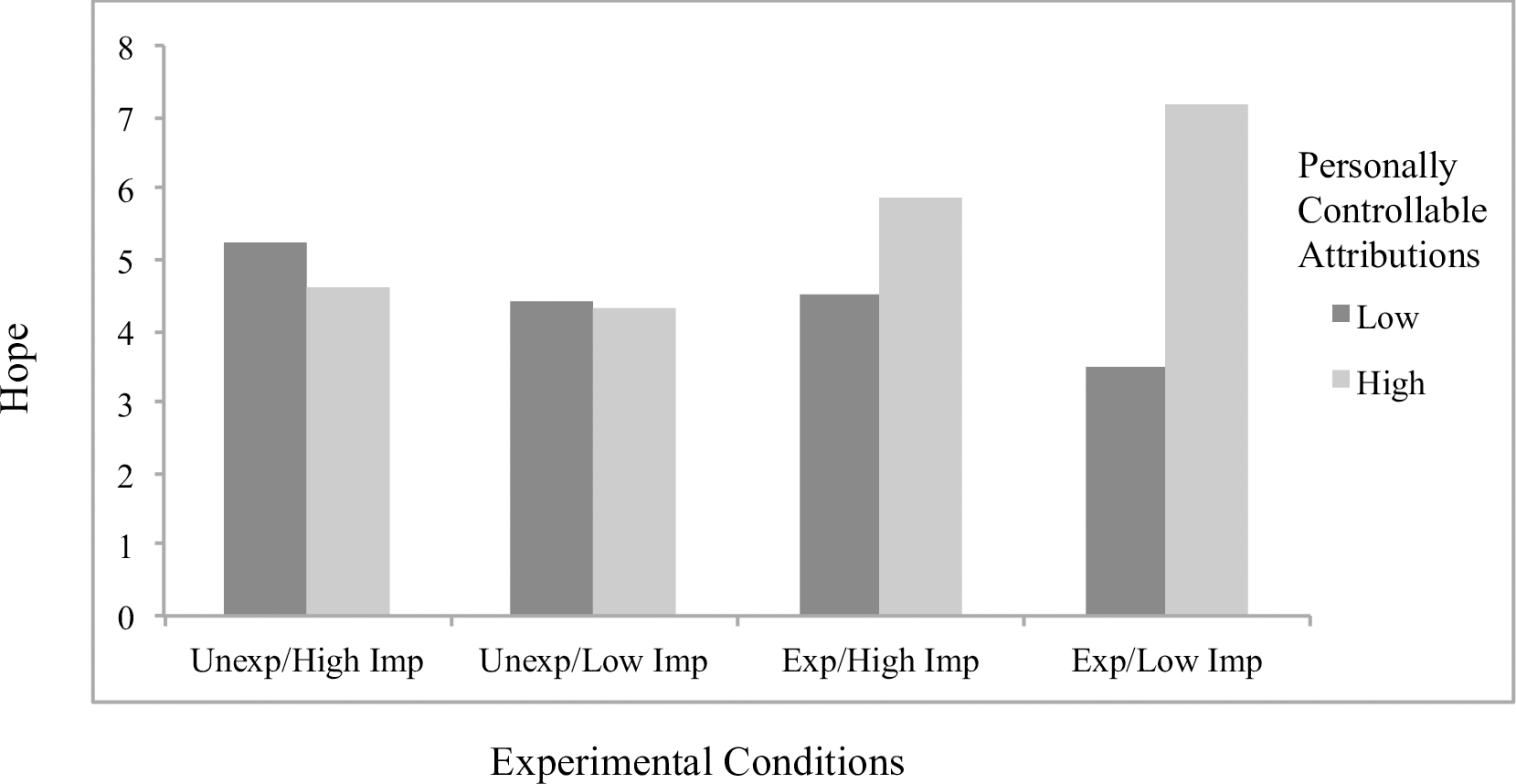
Effects of personally controllable attributions by experimental conditions on hope for online students.

**Fig 9:**
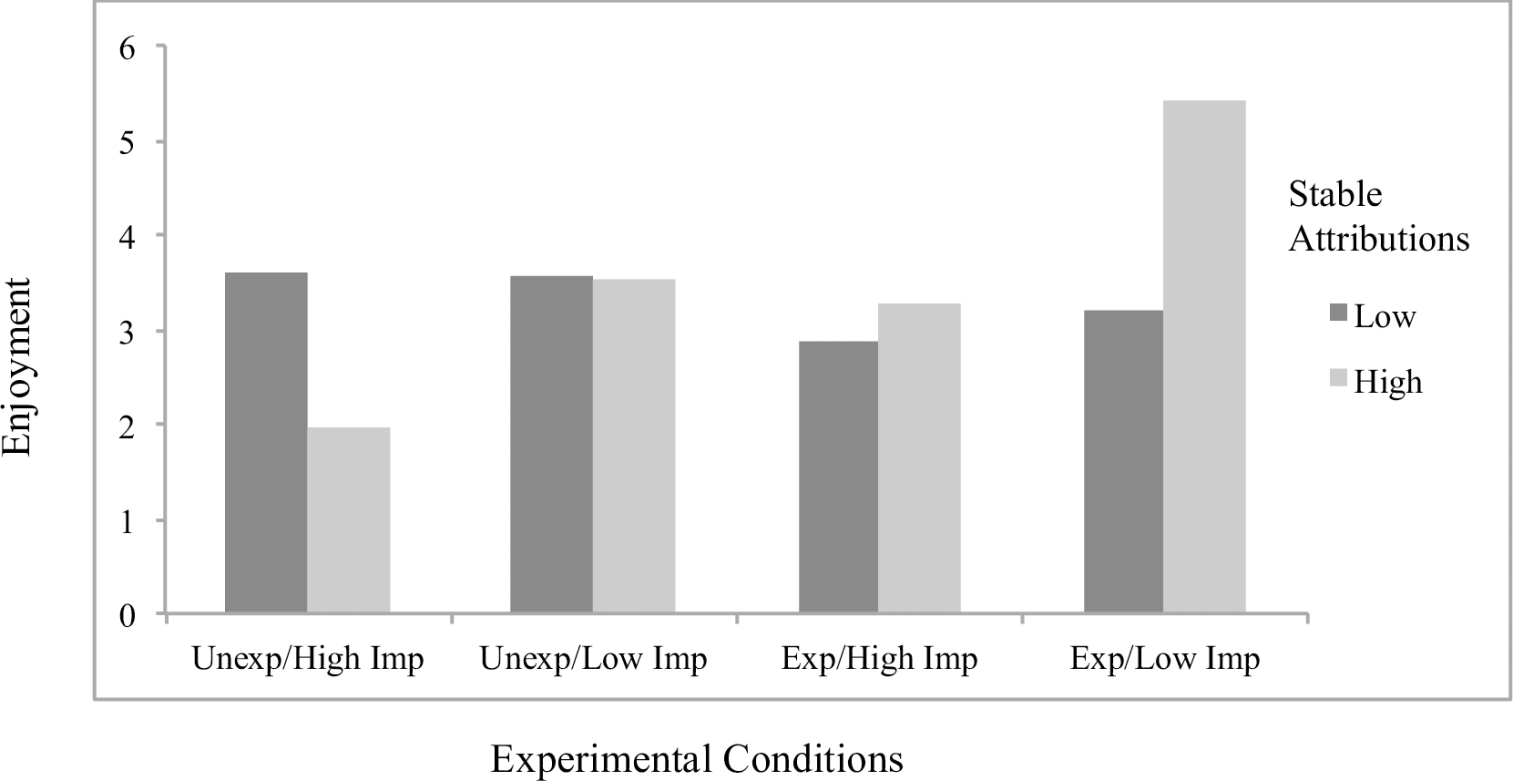
Effects of stable attributions by experimental conditions on enjoyment for online students.

Significant interactions further showed the effects of personally controllable attributions on hope to vary by experimental condition (Fig 8) specifically with respect to the *unexpected/high importance* (*B* = -.28, *p* = .004) and *unexpected/low importance* conditions (*B* = -.24, *p* = .007). Simple slopes analyses showed personally controllable attributions to predict greater hope only in the *expected/low importance* condition (*B* = .24, *p* = .001). Finally, a significant interaction showed stable attributions to have opposite effects on enjoyment depending on the type of computer problem experienced (*B* = -.39, *p* = .048; Fig 9), with simple slopes showing stable attributions to predict marginally lower enjoyment in the *unexpected/high importance* condition (*B* = -.16, *p* = .14) and marginally higher enjoyment for the *expected/low importance* condition (*B* = .22, *p* = .16).

### Study 2 Discussion

#### Hypothesis 1

Hypothesis 1 proposed that internal attributions for technology-related problems should predict more positive and less negative emotions concerning academic computing. In contrast to Study 1 showing internal attributions to predict less helplessness, the findings for Study 2 with respect to internal attributions were notably mixed. For online students, results in support of Hypothesis 1 were indeed observed showing internal attributions for computer problems to benefit positive emotions and predict less anxiety following serious events (unexpected, high importance). However, this hypothesis was contradicted with results for online students also showing internal attributions to predict *more* anxiety following non-serious computing failures (expected, low importance). In other words, whereas students in online programs found taking personal responsibility to help their anxiety if a computing problem was serious, taking responsibility for non-serious events predicted more anxiety. Findings for internal attributions also did not support Hypothesis 1 for traditional students who consistently reported *more* negative emotions if they reported taking personal responsibility for academic computing failures. Thus, although internal attributions were generally helpful for online students, they were consistently maladaptive for traditional students. With respect to externally controllable attributions, Study 2 findings were also contrary to Hypothesis 1 in showing these attributions to predict *lower* (not higher) anxiety, specifically for online students who experienced non-serious computing problems.

These unanticipated findings thus suggest that internal attributions can lead to negative emotions for typical students with less computing experience. Moreover, these results imply that internal attributions may be similarly detrimental for more experienced, online students when dealing with typical computer issues, with external attributions instead being more emotionally adaptive in such cases (see zero-order correlations showing more experience to correspond with more externally controllable attributions and enjoyment). Although these emotional benefits of externally controllable attributions for online students in Study 2 contradict both Hypothesis 1 and the disadvantages of these attributions for traditional students found in Study 1 (more guilt and anxiety following less serious problems), they nonetheless align with prior research suggesting possible benefits of external attributions for computer problems (e.g., more computer use, self-efficacy, knowledge [40]) and further suggest that these benefits might be specific to more typical vs. serious academic computing difficulties.

Concerning differences in effects for internal and externally controllable attributions between Studies 1 and 2, it is possible that these discrepancies might reflect students’ anticipated reactions to technological difficulties (Study 1, scenario method) as contrasted with their responses to actual computing problems (Study 2, experimental method). Whereas the emotional consequences of taking responsibility for computing difficulties may not be evident when considered abstractly (Study 1), more pronounced effects of internal attributions on anxiety may nonetheless become apparent following real-life computing events (Study 2). Similarly, although external explanations for computer problems may generally seem emotionally maladaptive when discussed hypothetically, or of imaginable benefit only in serious circumstances (Study 1), focusing on externally controllable reasons might in fact be emotionally beneficial when dealing with typical real-world computing challenges (Study 2).

Alternatively, these discrepant findings between Studies 1 and 2 may be due to underlying qualitative differences between traditional and online student populations. For example, given that online students tend to have more academic computing experience than traditional students, they might also have more varied explanations for regularly encountered computing problems that do not implicate their personal capabilities (e.g., software, hardware, network issues, etc.). As such, it is possible that internal attributions might be unnecessarily self-defeating for online students after non-serious computing setbacks, with attributions to external factors instead being a more beneficial response afforded by online students who tend to have more experience with these types of problems. Regardless of possible explanations, these findings clearly underscore the importance of assessing computing-related motivation and emotions both using experience-based methodologies as well as among students in asynchronous and traditional learning environments (for more on experience-based assessment, see [62]).

#### Hypothesis 2

Consistent with Hypothesis 2, personally controllable attributions for technology-related problems contributed to greater feelings of hope, specifically for online students in response to non-serious computer problems. However, most findings observed in Study 2 were directly contrary to Hypothesis 2, with traditional students generally experiencing *more* computing-related shame and anxiety if they focussed on personally controllable reasons for computer failure. Similar to the results for internal attributions (Hypothesis 1), whereas traditional students consistently found blaming computing setbacks on their own intentional behavior to be emotionally disconcerting, online students could experience limited emotional benefits from this type of attribution, namely if they focus on having personal control specifically over minor computing challenges. Once again, these findings largely contradict existing research on the emotional, motivational, and achievement benefits of personally controllable attributions typically observed in post-secondary students [50–51], suggesting not only that the typical assumptions of controllable attributions as adaptive may not directly apply to academic computing experiences, but also that traditional and online students may experience computing challenges differently and experience opposite effects of the same attributional approach.

#### Hypothesis 3

According to the third study hypothesis, stable attributions for technology-related problems were expected to predict less positive and more negative emotions concerning academic computing. Similar to Study 1, findings from Study 2 provided consistent support for this hypothesis in showing traditional students to experience more shame (regardless of problem severity) and more anxiety (concerning moderately serious problems) if they believed their computing challenges to be caused by stable factors that were unlikely to change over time. Although Study 2 also showed stable attributions to have mixed effects for online students depending on the type of computer problem (non-serious: more enjoyment; serious: less enjoyment), these effects were relatively weak and simple slopes tests failed to reach significance (*p*s ≤ .16). Accordingly, these findings provide support for Hypothesis 3 and existing research on causal attributions in post-secondary students showing stable attributions (e.g., lack of ability) to consistently predict more negative emotions, poor academic performance, and attrition as compared to unstable attributions (e.g., lack of familiarity [30,51]).

#### Hypothesis 4

Lastly, the effects of causal attributions on emotions were proposed in Hypothesis 4 to be most evident in the experimental condition in which the most serious computing problem was experienced (*unexpected*, *high importance*), with attribution effects being least evident in response to non-serious problems (*expected*, *low importance*). Although the benefits of internal attributions on anxiety, and risks of stable attributions for enjoyment, found in Study 2 are consistent with Study 1 (e.g., harmful stable attributions) and were indeed found for online students following the most serious computing problem, these post-hoc tests were only marginally significant and the remainder of the interaction effects observed were not consistent with Hypothesis 4. First, even though post-hoc contrasts for online students showing internal attributions to predict *more* anxiety, and stable attributions to predict *more* enjoyment, in the *non-serious* condition did not reach significance, this trend is nonetheless contrary to the assumption that the effects should be weakest for these students. Second, significant emotional benefits of external attributions and personally controllable attributions were in fact observed *only* for online students who experienced a non-serious problem. Third, the disadvantages of stable attributions for traditional students were found specifically concerning *moderately serious* problems (unexpected, but low importance) as opposed to more serious circumstances (unexpected, high importance). Thus, similar to Study 1, findings from Study 2 provide only partial support for Hypothesis 4 in indicating that although students’ attributions for computing challenges did impact their emotions differently depending on the type of problem experienced, significant attribution effects were observed following not only very serious events but also moderately serious and non-serious occurrences.

#### Limitations and open questions

Concerning the limitations of this second experimental study, it should be noted that the causal attribution and emotion measures did not include language specific to the manipulated technological difficulty to which the students were exposed, but instead evaluated their causal beliefs regarding a specific prior computing experience and their emotions in relation to academic computing problems more generally. This methodological decision was intended to preserve the believability of the manipulation (e.g., not have overly specific attribution items foreshadow a forthcoming glitch), and is consistent with existing research on general computing-related attributional profiles [41]. However, it is possible the lack of specificity otherwise obtained by assessing event-specific attributions immediately following the manipulated technological failure experience may have contributed to inconsistent or non-significant findings. Although the assessment of attributions and emotions thus warrants caution when interpreting results due to measurement limitations similar to those of Study 1 (i.e., generalization from specific situations), specific causal attributions for previous academic computing problems can be reasonably expected to inform emotions following problems within the same domain (i.e., academic computing; see prior research on domain-specificity of achievement emotions [57–59]).

An additional limitation concerns the experimental manipulation of perceived importance, which was operationalized as the loss of one paragraph as opposed to two paragraphs of text. Although the experimental manipulation followed from research identifying time lost due to computer problems as a primary reason for frustration in academic and occupational settings [60], future research investigating perceived importance of academic computing problems should evaluate more substantial manipulations of perceived importance (e.g., loss of one paragraph vs. an entire essay) and also include manipulation checks to ensure perceived importance was sufficiently induced. As it is possible that the difference in the amount of text lost between experimental conditions did not adequately elicit a discrepancy in perceived importance, the unanticipated findings regarding the importance dimension of causal search (e.g., stable attributions) should be interpreted with caution. Finally, despite the importance manipulation being intentionally moderate in nature so as to prevent study attrition and missing data on subsequent measures, significant participant attrition was observed in Study 2 with only 55% of the traditional sample and 67% of the online sample having completed the entire study. Although preliminary analyses found no significant differences as a function of attrition across multiple background variables (e.g., age, gender, program year, high school grades), the remaining sample could nonetheless differ from those who dropped out on variables not assessed in this study (as they did on stability attributions) thus warranting caution when interpreting results.

## General discussion

The present research aimed to investigate relationships between causal attributions and emotions specific to academic computing challenges encountered in post-secondary education. Each study presented these relationships utilizing in-depth self-report measures of attributional dimensions assessed with both traditional and online students, with Study 1 administering hypothetical scenarios and Study 2 utilizing experimental manipulations to further examine whether the type of computing problem experienced moderated attribution-emotion relationships. Findings from both studies show that students’ causal attributions do indeed significantly impact their emotions concerning technological challenges experienced in academic settings. Furthermore, the significant interaction effects observed provide empirical evidence that the relationships between students’ attributions and their emotions concerning computing difficulties are likely to be substantially affected by not only the type of computing problem but also their academic computing background (traditional vs. online post-secondary setting).

The first set of findings to consistently emerge from the present studies was the emotionally maladaptive nature of attributions for academic computing problems to stable factors, or conversely, the emotional benefits of attributions to factors that might change over time. Both Studies 1 and 2 showed the stability dimension to consistently predict more negative emotions, particularly in response to *unexpected* computer problems. This finding is not only consistent with considerable existing research based on Weiner’s [21] theory showing stable attributions to predict poorer academic outcomes [30,63], but also intervention studies showing how encouraging students to make unstable attributions for academic challenges can improve motivation and performance [64–65]. Accordingly, these findings clearly suggest that efforts to improve post-secondary students’ motivation and emotional experiences concerning academic computing should be most effective when they discourage attributions to factors that persist over time (e.g., limited computing ability) while instead promoting attributions for computing difficulties to factors that can change (e.g., suboptimal strategy, lack of familiarity).

A second set of findings observed in both studies suggested that internal and personally controllable attributions for computing challenges have mixed effects on students’ emotions. Whereas personally controllable attributions for academic setbacks are consistently beneficial for student learning, persistence, and achievement [44,66], with classroom-based motivational interventions often explicitly encouraging controllable attributions for academic failure (e.g., attributional retraining [25,29,67]), our findings showed that these benefits may not translate to academic computing contexts. Whereas internal and personally controllable attributions were found to be beneficial for traditional students in a hypothetical context (Study 1), they were also found to be detrimental for these students following real-life computing problems (Study 2). Similarly, although emotional benefits were observed following internal and personally controllable attributions for students in online programs (Study 2), these benefits varied significantly, or completely reversed, depending on the type of computer problem experienced. Overall, a consistent theme across Studies 1 and 2 is that internal and/or personally controllable explanations for computing difficulties can lead to very different emotional experiences depending on the specific attribution selected, method of assessment (scenario/experimental), academic context (traditional/online), as well the type of problem experienced, and should thus not be uniformly recommended as an adaptive response to technological challenges based on findings from other academic contexts.

Similarly, a third set of results to emerge suggests that, contrary to findings showing attributions to factors external to oneself to typically have a detrimental impact on academic outcomes [68–69], attributions to externally controllable factors may in fact be emotionally adaptive in response to certain types of academic computing problems. More specifically, externally controllable attributions were specifically found to predict lower levels of anxiety for traditional students in Study 1 (serious problems) as well as lower anxiety for students in online programs in Study 2 (non-serious problems). As these findings are consistent with studies with post-secondary students in non-achievement contexts showing external attributions to serve a self-protective function (e.g., interpersonal setbacks [54–55]), they suggest that assumptions concerning attribution-emotion relations based primarily on research in the achievement domain may similarly not be directly applicable to how students respond to technological challenges.

Concerning future directions, given the notably inconsistent effects of personally and externally controllable attributions as a function of hypothetical vs. experimental methods, traditional vs. online students, and/or type of computing difficulty, further research to replicate and better determine the conditional nature of these effects is recommended (for more on conflicting results from scenario vs. in vivo methods concerning causal search in university students, see [31]). Similarly, as significant interaction effects in Studies 1 and 2 showed stable attributions to be most maladaptive in response to unexpected (but not important) computing problems, further study on how to better approximate the element of importance in future scenario and experimental conditions may be needed to detect the proposed unexpectedness x importance interaction as per Weiner’s [21] theory [31,53]. Additionally, although no initial differences were found as a function of scenario condition in Study 1, initial differences in internal attributions and computing experience by experimental condition for traditional students observed in Study 2 warrant further investigation as to how problem importance and unexpectedness moderates effects of attributions on emotions in real-life contexts (e.g., observation, experimental methods). Accordingly, methodological improvements in future research could include baseline measures, longitudinal assessments, and a control group to provide stronger evidence of causal relations between study variables as well as more objective vs. self-report measures to improve ecological validity of findings (e.g., persistence on a computing task). Further, whereas face-valid, single-item emotion measures were considered more practical than composite scales so as to minimize the intrusiveness of our scenario and experimental protocols in Studies 1 and 2 (see [70]), future research utilizing multi-item emotion scales (e.g., AEQ [71]), is recommended to replicate our experimental condition effects with more intensive emotion assessments.

It should also be noted that despite the scenario measures (Study 1) and experimental methods (Study 2) having been developed in parallel (i.e., four levels of computing problem severity), results cannot be directly compared across methodologies due to differing technological difficulties being depicted (i.e., program used for class project crashing vs. loss of typed text online). It should also be noted that although the dimension-oriented measure of attributions employed in Studies 1 and 2 was adapted directly from an existing measure by McAuley et al. [43], and represents a more intensive multi-item assessment than measures that simply list causal attributions (e.g., ability, effort, luck; [29]), the effects pertaining specifically to stable attributions should be interpreted with caution due to low scale reliability. Concerning the emotion measures, as the emotions assessed were situation-specific in Study 1 (to the hypothetical scenario provided) and more general in Study 2 (concerning typical academic computing problems), this methodological difference may have contributed to the pattern of findings presented. Similarly, as participants’ reported emotions concerning the hypothetical scenarios in Study 1 required them to recall and extrapolate from similar personally experienced events, whereas Study 2 protocols employed a real-time computing failure, this methodological difference may also have contributed to findings for outcome-related emotions (e.g., guilt) being most evident in the scenario study, and effects in the experimental study being observed primarily for activity-related emotions (e.g., anxiety).

With respect to lacking study measures, the present findings also warrant more in-depth examinations of how students’ computing-related attributions influence not only their emotional experiences but other relevant academic outcomes such as learning, retention, and achievement so as to better inform the development of computing support interventions. Concerning participant recruitment, the present studies also included students from predominantly social science programs warranting future studies with students from across degree programs. Similarly, due to having recruited from only a single online institution (comprehensive) and one traditional university (research-intensive), future research in which multiple institutions of each type are contrasted is needed to replicate findings observed mainly for traditional students (Study 1) or for online students (Study 2). Finally, it should be noted that due to differing disciplinary affiliations of study participants between the online and traditional institutions, likely due to differing organizational structures (e.g., traditional sample: 23%-31% science students; online sample: 7%-10% science and technology students), further research with multiple institutions of each type is required to replicate the present differential findings as a function of institution type.

In sum, the present set of studies represents a critical initial application of Weiner’s [21] attribution theory to contribute to our understanding of how students’ emotional experiences with academic technology may be impacted by the way they interpret computing challenges. Computing-related attributions and emotions were investigated using both nonintrusive and intensive multi-item methods, as well as measures concerning both general academic computing and specific computing problems. The potential moderating influence of the type of computing problem students may face (four theoretically informed levels of severity) and their academic context (traditional vs. online settings) were also considered in examining the effects of computing-related attributions on emotions, with the study hypotheses further examined using parallel scenario and experimentally manipulated conditions. In this way, the second experimental study built upon the initial scenario study by way of improvements to protocols to provide a notably comprehensive examination of how students respond to and experience academic computing difficulties. It is anticipated that by providing a more in-depth understanding of students’ attributional beliefs and emotions concerning academic technology challenges that we can improve the educational support needed to help students respond to the ever-increasing demand for digital literacy in higher education.

## Supporting information

S1 Data Zipped folder file containing SPSS datasets for all studies. (ZIP)
